# From Mechanisms to Materials: Oximate Reactivity and Emerging Strategies for Organophosphate Detoxification

**DOI:** 10.1002/cphc.202500802

**Published:** 2026-04-19

**Authors:** Pedro Rodríguez‐Dafonte

**Affiliations:** ^1^ Departamento de Química Física Facultade de Química Universidade de Santiago de Compostela Santiago de Compostela Spain

**Keywords:** α‐effect, nanostructured, organophosphorus, oximes, supramolecular

## Abstract

The development of effective strategies for the detoxification of organophosphorus (OP) nerve agents has evolved from the early mechanistic studies of François Terrier and collaborators, who first elucidated the exceptional nucleophilicity of α‐effect species such as oximes and hydroxamates, to the modern design of supramolecular and material‐based systems. Terrier's pioneering kinetic investigations and the conceptual framework established by Clifford A. Bunton and Erwin Buncel on micellar catalysis provided a foundation for understanding how medium effects and local organization modulate α‐nucleophile reactivity. Building on these insights, contemporary research has expanded the chemical landscape of oxime‐based reactivators through synthetic modification, computational modeling, and the development of functional scaffolds capable of efficient acetylcholinesterase (AChE) reactivation and direct OP hydrolysis. This review examines the evolution of oxime‐based detoxification, with emphasis on structure–reactivity relationships, mechanistic insights, and advances in reaction media. Micellar systems were the first colloidal environments explored, while supramolecular assemblies such as lipids and cyclodextrins combine molecular recognition with catalytic function. Recent developments include inorganic and nanostructured catalysts that enable organophosphate degradation under mild conditions. The transition from α‐nucleophile chemistry to multifunctional materials reflects not only the progress of physical organic chemistry in detoxification but also its convergence with supramolecular and materials science.

## Graphical abstract

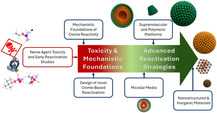

## 1 Introduction

Organophosphorus compounds (OPs) constitute one of the most toxic classes of synthetic chemicals ever developed, with dual use in agriculture as pesticides and in warfare and terrorism as nerve agents. Their mechanism of toxicity relies on the covalent inhibition of acetylcholinesterase (AChE), leading to a rapid accumulation of acetylcholine at cholinergic synapses and neuromuscular junctions. This results in a cholinergic crisis characterized by convulsions, paralysis, and ultimately respiratory failure if not rapidly treated [1, 2].

**SCHEME 1:**
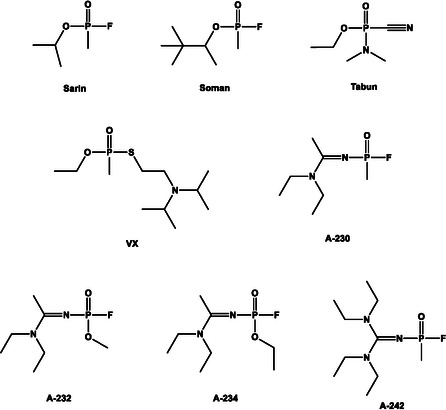
Nerve agents.

The first organophosphorus nerve agent, tabun, was synthesized in 1936 by Gerhard Schrader, followed shortly by sarin (1938) and soman (1944). Later, more persistent and lipophilic agents such as VX and the A‐series (Novichok) compounds were introduced (Scheme 1). Despite being banned under the Chemical Weapons Convention (CWC), organophosphorus nerve agents continue to pose a serious threat [3]. For clarity, the term organophosphates is used throughout this review in a broad sense to designate all organophosphorus nerve agents, irrespective of whether they are formally phosphate‐ or phosphonate‐derived. These compounds are characterized by their extremely high potency, diverse structural features, and their ability to induce covalent AChE phosphylation, leading to functionally irreversible inhibition unless rapidly counteracted.

The primary pharmacological strategy to counteract organophosphorus nerve agent poisoning is the reactivation of inhibited acetylcholinesterase (AChE) using nucleophilic agents, particularly oximes. Beyond classical oximes, recent studies have explored alternative nucleophilic strategies for OP detoxification. Hydroxamate‐based formulations have demonstrated rapid VX degradation and skin permeation compatible with within‐skin neutralization, supporting their use in topical ‘catch‐up therapy’ approaches. In parallel, hydroxamic acid salts have also been shown to promote fast hydrolysis of Novichok nerve agents, offering a simple and effective nucleophile platform that complements conventional reactivators [4, 5, 6].

**SCHEME 2:**
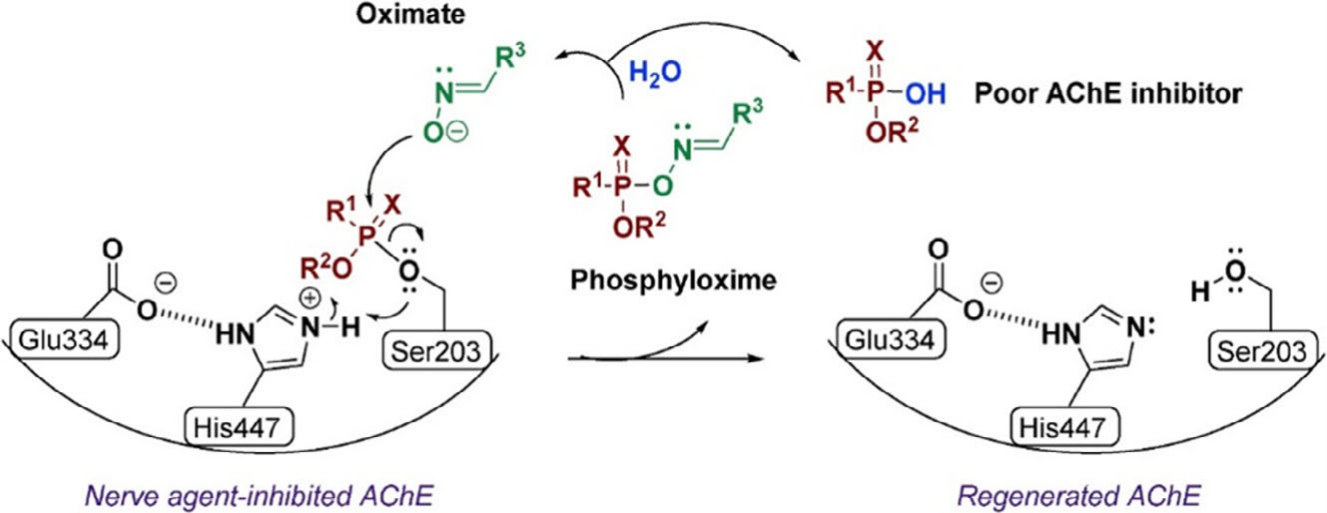
Inhibited AChE reactivated by an oximate (*X* = O, S). Reproduced from ref [3]. Licensed under CC‐BY 4.0.

**SCHEME 3:**
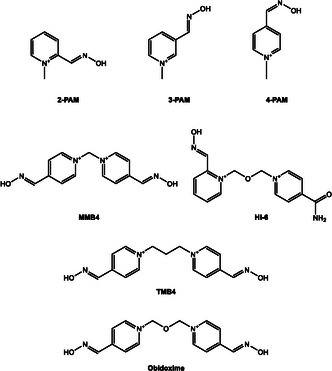
Classical oxime‐based antidotes employed since the early development of AChE reactivators.

Oxime compounds target the phosphylated serine residue at the enzyme's active site, promoting the release of the organophosphorus group and restoring enzymatic function (Scheme 2). Oximes are favored due to their high α‐nucleophilic character and established efficacy under physiological conditions. The first oxime reactivator, 2‐PAM (2‐[(hydroxyimino)methyl]‐1‐methylpyridinium) (see Scheme 3), was discovered by Wilson in 1955 and remains in use today in both military and civilian contexts [7]. Notably, it is currently the only oxime reactivator approved by the United States Food and Drug Administration (FDA) [8]. Despite intense research efforts, no alternative functional group has consistently demonstrated superior reactivation efficiency toward OP‐inhibited AChE. The high reactivity of oximes is attributed to their α‐effect, arising from the adjacent lone pair that enhances nucleophilic attack at the phosphorus center. This effect is mechanistically illustrated in Scheme 2, which depicts the nucleophilic reactivation of phosphylated AChE by an oximate anion, leading to enzyme regeneration and release of a phosphoryloxime by‐product. It is important to note that reactivation must occur before the phosphylated enzyme undergoes aging, a spontaneous dealkylation process that renders the adduct refractory to nucleophilic attack, particularly relevant in soman poisoning. Moreover, the phosphoryloximes produced upon reactivation retain toxic potential, as they can re‐phosphylate AChE.

The marked nucleophilic reactivity of oximes is generally ascribed to their classification as α‐nucleophiles, species bearing a vicinal heteroatom lone pair whose reactivity cannot be rationalized by basicity alone [9]. This anomalous behavior, commonly referred to as the α‐effect, has long fascinated physical organic chemists, though the mechanistic basis eluded definition for many years. Establishing the conditions of structure and medium under which oximes would display optimal behavior required that its mechanism be investigated in detail, especially in chemically controlled systems. In these respects, the works of Terrier have been particularly seminal, providing a general and mechanistically sound conceptual framework that underpins interpretation of the reactivity of oximes toward electrophilic phosphorus. Over a period of over thirty years, Terrier systematically deconstructed the structure‐basicity‐medium‐nucleophilic efficiency relationships, stating the basic principles that underpin the rational design of modern reactivators.

One of Terrier's latest publications in this line of research is a book chapter, published in 2009 [10], which summarizes the most significant advances in the role of oximates as α‐nucleophiles in dephosphylation reactions. The chapter is of considerable historical significance, offering a retrospective overview of Terrier's own contributions. Coauthored with Buncel and Um, the chapter examines the nucleophilic behavior of hydroxylamines, oximates, and hydroxamates, with particular emphasis on their function as α‐nucleophiles in substitution reactions involving electrophilic centers of carbon (C), phosphorus (P), and sulfur (S). This work represents one of the latest collaborations between Terrier and Buncel and summarizes part of the joint research they carried out over such a long period of time. Both professors continued publishing until 2017, but the chapter referenced here brilliantly summarizes the most important aspects of their joint research on α‐nucleophilicity. Terrier and Buncel passed away in 2024 and 2018, respectively, leaving behind extremely important work in the field of organic physical chemistry. Solvent composition plays a pivotal role in shaping α‐nucleophilicity, with bell‐shaped kinetic profiles and reversed Brønsted correlations frequently observed, attributed to differential solvation and desolvation processes. The chapter also examines the influence of micellar systems as reaction media and introduces novel detergent architectures incorporating oxime or hydroxamate moieties into imidazole rings, aimed at facilitating targeted dephosphylation reactions. Taking the content of the chapter as a reference, we will now briefly review some of the main contributions made over the years by Terrier's group in the study of oxime reactivity.

In 1988, five new oximes were proposed and kinetic analyses showed that they exhibited high reactivity. In complement to this, low toxicities were observed in in vitro experiments, which may form the basis of their practical application [11]. Subsequently, Terrier's research group demonstrated a practical and mechanical advance by using a fluoride ion selective electrode to monitor the real‐time hydrolysis of nerve agents (sarin, soman, and diisopropylphosphorofluoridate (DFP)) in aqueous media oxime‐mediated by nucleophiles [12]. The study demonstrated that, under appropriately buffered conditions, oximates were capable of achieving complete and rapid hydrolysis of organophosphorus agents, even in reactions with half‐lives as short as 30 s. These excellent results highlighted the viability of kinetic studies in toxicologically relevant systems and confirmed the high detoxifying efficacy of oxymates against nerve agents.

**FIGURE 1:**
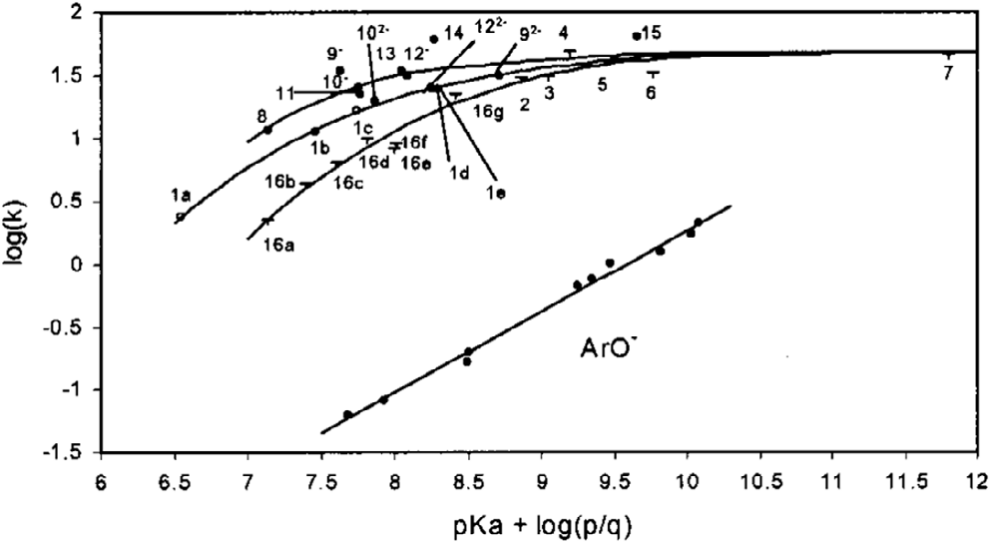
Brønsted‐type nucleophilicity plots for reactions of oximates and phenoxides (ArO^‐^) with PNPA at 25°C in aqueous solution. Reproduced from ref [13] with permission from American Chemical Society. Copyright 2002.

**SCHEME 4:**
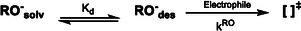
Reaction mechanism. Scheme created by the author based on ref [9].

In their 2002 communication Terrier and Buncel [13] explored the α‐effect in more fundamental terms. Using oximate nucleophiles in reactions with the carbonyl substrate p‐nitrophenylacetate (PNPA), the authors observed a plateau in reactivity beyond a pK_a_ of approximately 8 (see Figure 1). The leveling‐off observed at high pK_a_ values arises from a substantial decoupling between nucleophile desolvation and bond formation. This ‘solvational imbalance’ penalizes the reactivity of highly basic oximates in water. However, when the solvent is switched to Me_2_SO‐rich mixtures, this imbalance is mitigated, restoring linear Brønsted correlations and reestablishing the α‐effect. The reaction mechanism is shown in Scheme 4.

An explanation for the behavior emerges from further consideration of solvational imbalance models proposed by Jencks and Bernasconi [9, 14]. Taking the straight Brønsted line with slope *β*_nuc_ defined by the low‐pK_a_ data points, as a reference, and assuming that desolvation occurs in a pre‐equilibrium step (*K*_d_ < 1) preceding nucleophilic attack, the curvature observed in Brønsted plots at higher basicity can be interpreted as negative deviations arising from increased desolvation costs. These deviations are quantitatively accounted for by Equation (1).

(1) $$\text{Δ}\log\left(k^{\mathrm{RO}}\right)=\left(1-\beta_{\mathrm{nuc}}\right)\log K_{d}$$

This clearly demonstrates that as desolvation becomes increasingly energetically demanding, the progressively more negative log (*K*_d_) values lead to correspondingly lower $\text{Δ}\log(k^{\mathrm{RO}})$ values, accounting for the observed leveling‐off in reactivity under strongly aqueous conditions.

Building on this, the group turned to phosphorus electrophiles in a 2003 study [15], seeking to determine whether a similar leveling‐off behavior occurred in reactions directly relevant to organophosphorus detoxification. Their results showed that the extent of reactivity saturation varied depending on the nature of the phosphorus center. The study examined the reactivity profiles of two model organophosphorus esters: bis(4‐nitrophenyl) methyl phosphonate (BNPMP) and bis(4‐nitrophenyl) phenyl phosphonate (BNPPP). In addition, the widely used insecticide diisopropyl fluorophosphate (DFP) was also used in the kinetic study. While DFP and BNPMP exhibited a leveling‐off in reactivity at pK_a_ values above 9–9.5, BNPPP displayed a bell‐shaped profile, with maximum reactivity followed by a marked decline in rate (*β*_nucl_ < 0) within the same pK_a_ range. These contrasting behaviors highlight the role of electrophilic structure in modulating the expression of the α‐effect. A plausible explanation for the observed experimental differences is that the phenyl group in BNPPP introduces greater steric hindrance to the approach of the oximate nucleophile toward the phosphorus center, in comparison with the smaller methyl group present in BNPMP. Considering that the most basic oximates are also the most extensively solvated, the energetic penalty associated with desolvation prior to nucleophilic attack would be more pronounced in the case of PNPPP, thereby restricting its reactivity at higher pK_a_ values. This interpretation accounts for the observed decrease in reactivity of BNPPP under such conditions and reinforces the notion that the α‐effect is not an intrinsic property of the nucleophile alone but is modulated by the structure of the electrophile and the surrounding medium.

Together, these studies laid the foundation for the group's most comprehensive investigation, published in 2006 [16] in Organic & Biomolecular Chemistry. There, Terrier’ group studied the kinetics of oximate reactions with various phosphorus electrophiles: BNPPP, BNPMP, sarin, soman, and DFP. Experimental data once again reveal curvature in Brønsted‐type nucleophilicity plots for oximate reactions with esters. This behavior can be reasonably explained by the earlier hypothesis that strongly hydrogen‐bonded species, such as oxyanions, must undergo partial desolvation before participating in nucleophilic attack. As basicity increases, the energetic cost of desolvation rises due to enhanced solvation of highly basic anions. The resulting disparity between the degrees of desolvation and bond formation at the transition state, commonly referred to as an imbalance [14, 17, 18], was extensively discussed. However, accumulated evidence suggests that a more general model is needed, one that treats both processes as occurring within the same step, but with desolvation progressing further than bond formation. Equation (2) describes the decrease in $\log(k^{\mathrm{RO}})$.

(2) $$\text{Δ}\log\left(k^{\mathrm{RO}}\right)=(\alpha_{\mathrm{des}}-\beta_{\mathrm{nuc}})\log K_{d}$$

Equation (2) defines $\alpha_{\mathrm{des}}$ as a parameter ranging from 0 to 1, representing the degree of desolvation achieved in the transition state. Notably, Equations (1) and (2) convey a consistent mechanistic insight: the emergence of curvature in Brønsted‐type plots due to solvation effects requires that partial desolvation of the nucleophile becomes increasingly energy‐intensive with rising pK_a_ and that this desolvation precedes bond formation along the reaction coordinate. The study confirmed and extended previous findings, demonstrating that the Brønsted correlation for oxime reactivity plateaus at high pK_a_ values. This leveling‐off is established as an intrinsic property of the oximate functionality, reflecting a saturation effect that arises from energy imbalances between the desolvated nucleophile and the transition state. As basicity increases, desolvation becomes increasingly energy‐intensive, yet this process must still precede bond formation for effective nucleophilic attack. Consequently, any expected improvement in kinetic efficiency derived from the use of more basic oxymates will tend to be negated in aqueous solutions with pK_a_ values above 8–9. Moreover, substrate identity, solvation dynamics, and steric factors further modulate the reaction outcome, underscoring the multifactorial nature of oximate reactivity.

Grounded in this mechanistic understanding, it becomes clear that the challenge is not only to design more potent oximes but also to develop formulations and delivery strategies that can overcome pharmacokinetic and physiological barriers. Oximes continue to serve as the cornerstone of antidotal therapy against organophosphorus (OP) poisoning, although their clinical efficacy is still constrained by inherent physicochemical limitations. Classical pyridinium aldoximes, such as 2‐PAM, obidoxime, TMB‐4, and HI‐6, are effective against specific nerve agents but fail to offer broad‐spectrum protection, particularly against compounds like soman and tabun. Moreover, their high polarity and unfavorable lipophilicity hinder penetration across the blood–brain barrier (BBB), compromising their ability to protect the central nervous system [19]. The present review is structured into three main thematic areas:

• A mechanistic exploration of oxime reactivity, focusing on kinetic behavior and the α‐effect.
• The identification of structure–reactivity relationships and the rational design of molecular scaffolds.
• The investigation of alternative media and nanomaterial‐based systems that modulate nucleophilic performance and enhance drug delivery strategies.

The aim of this review is to highlight the most significant contributions from the past 15 years that align with Terrier's line of research. It does not seek to provide an exhaustive account of all studies on the chemical reactivity of organophosphates with oximes or related compounds, as such a task would be far too extensive for a single article. Instead, the focus is on offering a curated overview of research that, in our view, has been directly or indirectly shaped by Terrier's earlier work.

## 2 Mechanistic Foundations of Oxime Reactivity toward Organophosphorus Compounds

The kinetic studies conducted by Terrier and his collaborators laid the experimental groundwork for understanding the special reactivity of oximes. Their work established oximes as α‐nucleophiles and highlighted the special nature of their reactivity. On this basis, subsequent research has continued to explore the reactivity of oximes, particularly their role as preferred nucleophiles toward electrophilic phosphorous centers, thereby expanding our understanding of their versatility and potential for catalytic behavior. As anticipated in the introduction of this article, oximes exhibit nucleophilic reactivity that exceeds predictions based on their intrinsic basicity, a phenomenon traditionally attributed to the α‐effect. Although the involvement of lone pairs of adjacent heteroatoms has been proposed to explain the increased stabilization of the transition state and the reduction of activation barriers, the α‐effect remains ambiguous from a mechanistic point of view. In oximes, this effect was shown to depend largely on solvation, molecular orientation, and the nature of the electrophile. This suggests that oximes are a valuable model for investigating the factors governing α‐nucleophilicity.

A systematic experimental contribution to the understanding of the α‐effect came from Ik‐Hwan Um and co‐workers, who extended previous mechanistic framework by combining linear free‐energy relationships (LFERs), Brønsted analyses, and Yukawa–Tsuno correlations across a broad spectrum of nucleophiles [20, 21, 22, 23, 24, 25]. Among the studies most directly relevant to the mechanistic understanding of organophosphorus compound decontamination, the joint contributions of Um and Buncel are particularly noteworthy. In a particularly insightful study, they explored the influence of alkali‐metal cations on the nucleophilic displacement of authentic organophosphorus (OPs) substrates, specifically paraoxon and parathion, by ethoxide, providing valuable insight into ion‐pairing effects and transition state modulation [26]. This work revealed that reactivity at the phosphorus center depends not only on the nucleophile itself but also on the nature of the electrophilic core (*P* = O vs. *P* = S) and its interaction with the counter‐cation. While LiOEt was markedly more reactive than NaOEt or KOEt for paraoxon (*P* = O), the opposite trend was observed for parathion (*P* = S), with Li^+^ even exerting an inhibitory effect. The so‐called ‘thio effect’ emerged as a central theme: *P* = O substrates were intrinsically 50 times more reactive than *P* = S analogs toward EtO^‐^, and this difference grew to over 2000‐fold under Li^+^ coordination. A cyclic four‐membered transition state involving simultaneous nucleophile–cation–substrate interactions was proposed, illustrating how external medium effects and ion pairing can decisively modulate OP hydrolysis.

Building on their earlier work, Um and Buncel extended their studies to more typical α‐nucleophiles. In a subsequent article, they investigated the reactions of O‐p‐nitrophenyl thionobenzoate (PNPTB) with hydroxide, p‐chlorophenoxide (a conventional nucleophile) and butan‐2,3‐dione monoximate (an α‐nucleophile), in mixed DMSO/H_2_O solvents of varying composition [27]. By comparing these reactions with those involving p‐nitrophenyl benzoate, they assessed how replacing a C = O group with a more polarizable C = S bond affects reactivity and the α‐effect. Their findings show that nucleophilic substitution involving α‐nucleophiles is strongly influenced by both the polarizability of the electrophilic center and the solvent environment. Crucially, the α‐effect is not governed solely by ground‐state desolvation, but also by transition‐state stabilization (particularly in DMSO‐rich media) underscoring the combined impact of solvent and electrophile structure on α‐nucleophilicity.

**FIGURE 2:**
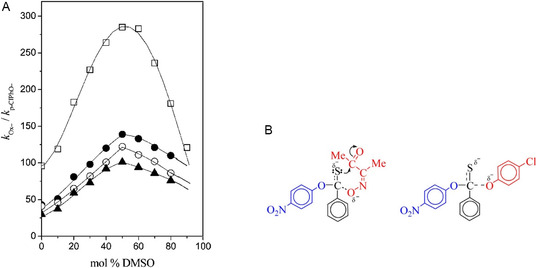
(A) Plots of the magnitude of the α‐effect (k_Ox_^−^/k_p‐__ClPhO_^−^) vs mol % DMSO for the reaction of PNPTB with Ox^−^ and p‐ClPhO^−^ at 15.0 (•), 25.0 (○), and 35.0°C (▴) and PNPA at 25.0°C (□). (B) Transition‐state (TS) structures for the reactions with Ox^–^ (a six‐membered cyclic TS) and p‐ClPhO^–^ (an acyclic TS). Reproduced from ref [28], with permission from American Chemical Society. Copyright 2013.

Expanding upon these findings, the subsequent collaborative study advanced the mechanistic understanding by quantitatively dissecting the activation parameters [28]. This work reveals that the bell‐shaped α‐effect observed in the nucleophilic substitution of PNPTB by oximate and reference phenoxide nucleophiles in DMSO–H_2_O mixtures is primarily governed by differences in the entropy of activation (TΔS^‡^), rather than enthalpy (ΔH^‡^). The α‐effect increases with DMSO content up to 50%, then decreases (Figure 2a), and this behavior is attributed to the contrasting transition state structures: a cyclic transition state for the oximate and an acyclic one for phenoxide (Figure 2b). The results highlight that the solvent‐dependent α‐effect is controlled by the entropy term, challenging the traditional view that enthalpy dominates, and emphasize the importance of transition state structure and solvation in modulating nucleophilic reactivity.

Complementing the mechanistic insights from previous studies, the investigation by Um and Han further refines our understanding of the α‐effect in nucleophilic substitution at phosphorus centers [29]. By comparing oximate and phenoxide nucleophiles reacting with aryl diphenylphosphinates in mixed DMSO/H_2_O media, the study confirms a concerted mechanism and reveals that the α‐effect is modest and independent of the leaving group's basicity. Crucially, it attributes the origin of the α‐effect to ground‐state desolvation of the oximate, rather than transition‐state stabilization, an interpretation supported by the small *β*_nuc_ values and the absence of intramolecular hydrogen bonding in the transition state. This work reinforces the idea that solvation dynamics and nucleophile structure play a central role in modulating reactivity, and it complements the entropy‐driven explanation proposed previously by highlighting the contribution of ground‐state energetics. Taken together, these four studies [26, 27, 28, 29] offer a detailed and complementary view of α‐nucleophile reactivity, demonstrating that the α‐effect arises from a nuanced interplay of ground‐state desolvation, transition‐state structure and solvent modulation. Experimental kinetic studies alone were insufficient to resolve these questions or fully characterize the underlying mechanisms. This limitation prompted the use of theoretical and computational approaches to explore reaction pathways and transition states in greater molecular detail.

**SCHEME 5:**
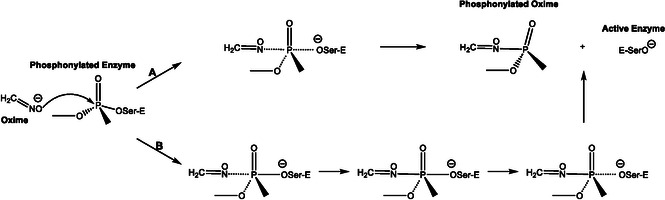
Reaction mechanism. Scheme created by the author based on ref [30].

In this context, the theoretical study by Wang and co‐workers marked a decisive advance by translating the experimental insights of Terrier, Um, and Buncel into explicit molecular pathways. Their computational work provided a detailed, atomistic understanding of the oxime‐induced reactivation of sarin‐inhibited acetylcholinesterase, bridging the gap between kinetic observations and the underlying reaction mechanism [30]. This theoretical study uses density functional theory (DFT) and the Møller‐Plesset perturbation theory to elucidate the mechanism by which oximate anions (specifically formoximate) reactivate acetylcholinesterase (AChE) that has been inhibited by the nerve agent sarin. As is typical for nucleophilic substitution, this reaction can proceed by two possible pathways: either as a one‐step process where nucleophilic attack and serine departure occur simultaneously or as a two‐step process involving formation of a pentacoordinated intermediate before serine is released (see Scheme 5). The results revealed that the reactivation proceeds via a mainly two‐step process. First, the oximate nucleophile attacks the phosphorus atom of the sarin–AChE adduct, forming a relatively stable pentacoordinated intermediate; second, the active serine residue is regenerated through an elimination step. Both steps are competitive and characterized by low energy barriers (around 6 kcal/mol), indicating that the reaction can occur rapidly and efficiently. The study also shows that solvation, modeled by polarizable continuum model (PCM), does not significantly hinder the process, supporting the potential of oximate anions as effective antidotes for sarin‐inhibited AChE. This work provides explicit molecular‐level insight into the reactivation pathway, bridging experimental observations with detailed mechanistic understanding.

Furthering this mechanistic perspective, Delfino and Figueroa‐Villar [31] extended the computational analysis to nucleophiles that are structurally analogous to the formoximate: the acetaldehyde enolate, methylperoxile, methylsulfenate, formaldehyde hydrazonate, and the neutral formoxime. The authors conclude that each nucleophile reactivates the sarin‐inhibited serine through broadly similar mechanisms, distinguished primarily by the orientation of the antidote in the initial step of the reaction pathway. While confirming Wang's central insight that reactivation proceeds via pentacoordinated phosphorus intermediates accessible through both concerted and stepwise pathways, the study emphasized the critical influence of nucleophile structure on the reaction mechanism. Formoximate followed a three‐step pathway, whereas the other nucleophiles proceeded through four steps. For formoximate, the first transition state involves a concerted nucleophilic attack coupled with serine rotation. In contrast, for the other nucleophiles, these events occur in separate steps. The nucleophilic attack is followed by the formation of an intermediate (absent in the formoximate pathway) after which the serine undergoes rotation. Notably, the α‐effect did not emerge as a decisive factor in these nonenzymatic reactions, as energy barriers were comparable across nucleophiles regardless of its presence. Hydrazonates emerged as promising candidates due to their favorable energy profile and stability, unlike peroxyl and sulfenate species, which were less viable physiologically. The article confirmed that the deprotonated form of oximes (oximate) is markedly more reactive than the neutral form, supporting its formation within the AChE active site. These results advance the mechanistic understanding of AChE reactivation and point to hydrazonates as antidotal candidates for further testing, with hydrazonate standing out for its favorable energy profile and chemical stability, unlike peroxyl and sulfenate species, which appear less viable under physiological conditions.

**SCHEME 6:**
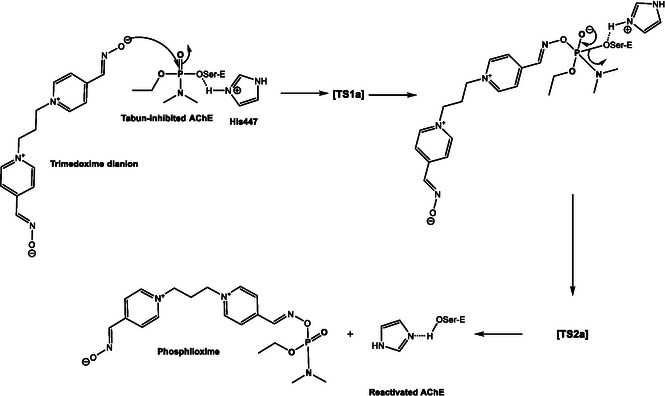
Reaction mechanism. Scheme created by the author based on ref [32].

Subsequent computational studies further diversified the range of mechanisms and refined the options available for understanding in detail the decontamination of organophosphorus nerve agents using oximes or similar nucleophiles. Lo and Ganguly [32] used DFT and ab initio calculations to study the reactivation of tabun‐conjugated AChE by neutral pyridinylhydroxylamine, a newly designed nucleophile, and compared its efficacy with various bispyridinium reactivators. In all cases, both with neutral and charged compounds, reactivation followed an addition–elimination mechanism: the nucleophile attacks the phosphorus of the tabun–serine complex, forming a trigonal bipyramidal intermediate that then dissociates into the phosphylated oxime and the reactivated enzyme. For charged oximes, the second transition was identified as the rate‐determining step (see Scheme 6). The results indicate that the activation barrier of neutral pyridinylhydroxylamine in the rate‐determining step is substantially lower than that of the charged oximes previously identified in the literature as the most kinetically effective antidotes against tabun‐inhibited AChE. However, neutral drugs exhibit weaker interaction energies with AChE compared to charged oximes, which facilitates the latter's movement within the active‐site gorge and suggests they may be structurally more suitable for reactivating tabun‐inhibited AChE. Steered molecular dynamics (SMD) simulations identified key interaction features, such as hydrogen bonds and hydrophobic contacts, for both the newly designed neutral oxime and the charged oxime TMB4. Notably, TMB4 undergoes full rotation of its pyridinium ring along the gorge axis during dissociation, moving away from the active site, whereas the neutral candidate remains oriented toward the inhibited serine. Additionally, lipophilicity/hydrophilicity calculations supported BB permeability assessments. Toxicity and AChE affinity data suggest that pyridinylhydroxylamine exhibits a toxicity profile comparable to that of established charged and neutral antidotes. This work broadened the focus from charged oximates to neutral analogs, anticipating the importance of pharmacokinetic considerations alongside reactivity.

**FIGURE 3:**
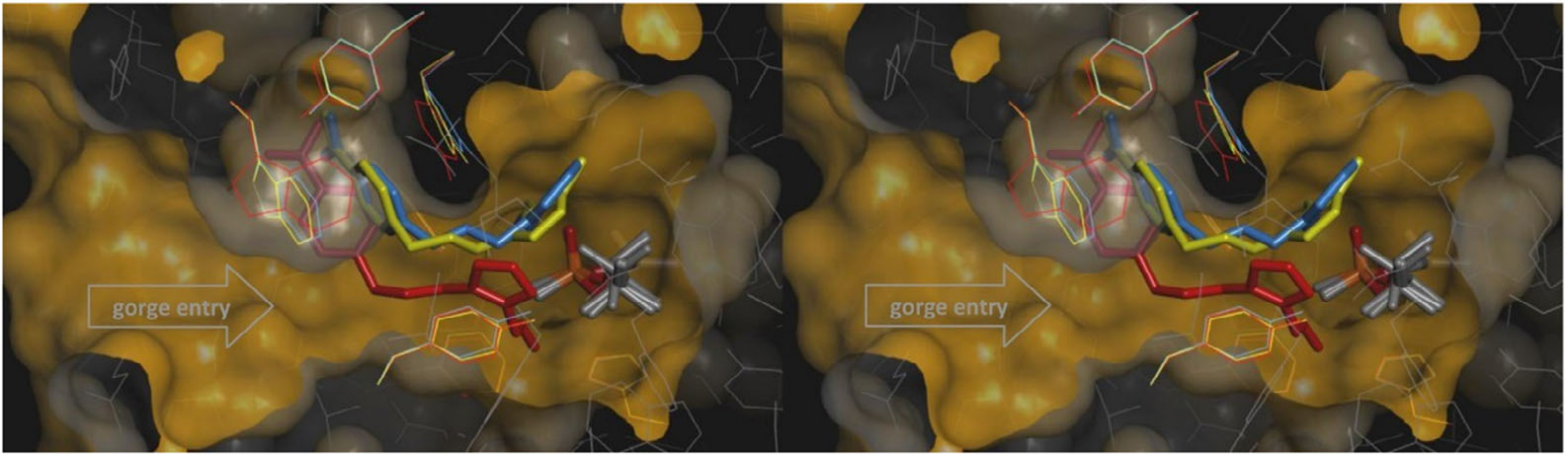
Stereo representation of computational molecular models of oximes RS‐170B (red sticks), HI‐6 (blue sticks), and RS‐169A (yellow sticks) bound reversibly to soman inhibited human AChE mutant Y337A/F338A represented by the orange Connolly surface and side chains as lines. Side chains allowed to rotate during simulation were colored after the corresponding oxime, and all other side chains are represented as gray lines. Atoms of phosphonyl Ser203 are represented by sticks, P (orange), C (gray), and O (red). The most optimally positioned conformers out of 10 calculated conformers for each oxime are shown. A monoquaternary, imidazole aldoxime N‐propylpyridinium, RS‐170B binds noticeably deeper in the gorge, as compared to the bisquaternary imidazolium aldoxime N‐propylpyridinium, RS‐169A and bisquaternary bispyridinium aldoxime HI‐6, while maintaining proper reaction geometry for an SN2 attack on the phosphorus. Reproduced from ref [33], with permission from American Chemical Society. Copyright 2025.

**SCHEME 7:**
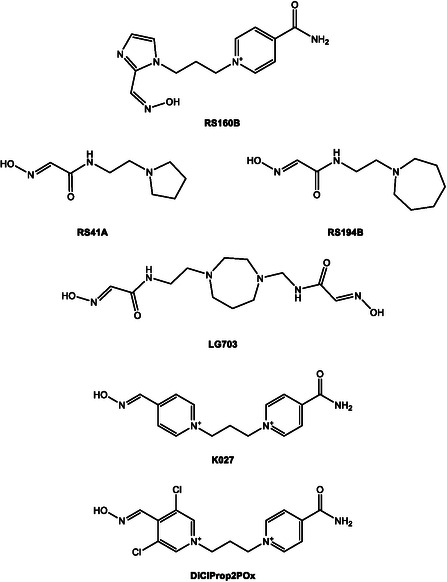
Structures of selected oxime reactivators commonly investigated for AChE reactivation.

After reviewing some of the fundamental mechanisms of oxime‐mediated dephosphylation at the molecular level, subsequent computational and hybrid experimental‐theoretical studies have focused on how these processes unfold within the complex environment of acetylcholinesterase, where orientation, solvation, and dynamic activation critically modulate the efficiency of reactivation. One of the most active current lines of research focuses on the administration of human enzymes or plasma components capable of neutralizing OP compounds before they interact with their biological target, AChE. These bioscavengers include stoichiometric agents such as plasma butyrylcholinesterase, which covalently bind OPs, and engineered AChE mutants with pseudocatalytic activity that, when assisted by oximes, can degrade OPs. In a predominantly experimental study, Kovarik and colleagues assessed the bioscavenging potential of various oximes through in vitro, ex vivo, and in vivo approaches, aiming to demonstrate the concept of oxime‐assisted catalytic scavenging of soman [33]. The researchers incorporated molecular dynamics simulations to investigate how different oximes are positioned within the active‐site gorge of acetylcholinesterase (AChE). Their findings revealed that RS‐170B (red sticks in Figure 3), an imidazole aldoxime (Scheme 7), adopts a more favorable geometry than the clinically established HI‐6 (blue sticks in Figure 3). In the case of RS‐170B, its geometry enables an in‐line S_*N*_2 reactivation by effectively accommodating the sterically hindered active‐site gorge of the organophosphate–AChE complex. These findings highlight the importance of steric compatibility, hydrogen‐bonding networks, and prereactive alignment within the enzymatic environment.

Pathak and Bandyopadhyay [34, 35, 36] focused on oximes and their fluorinated analogs and studied the unbinding transition of the oxime from the active‐site gorge of acetylcholinesterase (AChE) using molecular dynamics simulations with the effective polarization in the mean‐field polarizable (MFP) model of TIP3P water. The use of this type of oxime is based on the fact that fluorine increases drug lipophilicity, enhancing its ability to cross the blood–brain barrier. This makes fluorinated compounds promising candidates for reactivating AChE inhibited by organophosphates in the central nervous system. However, the molecular interactions between these drugs and proteins require further investigation. For HI‐6 and FHI‐6, the article elucidates the transition states and energy barriers associated with the nucleophilic attack, emphasizing the critical role of water polarization in the molecular dynamics of the protein–ligand complex [36]. Including this parameter proved essential in an otherwise effective polarizing environment, which typically only accounts for ionic charge rescaling of the substrates. Building on this, the authors examined the unbinding of fluorinated obidoxime (FOBI) versus its nonfluorinated counterpart (OBI) [34]. As in previous cases, the results suggest that fluorinated compounds may offer superior reactivation efficiency.

The development of more effective reactivators has also benefited from a systematic interplay between computational modeling and experimental validation. In a noteworthy medicinal chemistry effort, Kovarik’ group synthesized and evaluated a series of chlorinated bispyridinium mono‐oximes, specifically designed to lower the pK_a_ of the oxime group, thereby increasing the proportion of the active oximate form at physiological pH [37]. Complementary molecular modeling and permeability assays supported the kinetic reactivation data, revealing that dichlorinated analogs achieved 3–8‐fold improvements in reactivation efficiency against sarin‐, VX‐, and cyclosarin‐inhibited human AChE compared to the parent oxime. Computational docking and recognition studies further demonstrated that enhanced binding affinity and favorable orientation within the active‐site gorge were key contributors to this improved performance. The combined effects of reduced basicity, increased membrane permeability, and maintained low cytotoxicity underscore halogen substitution as a rational and promising design strategy for next‐generation AChE reactivators.

**FIGURE 4:**
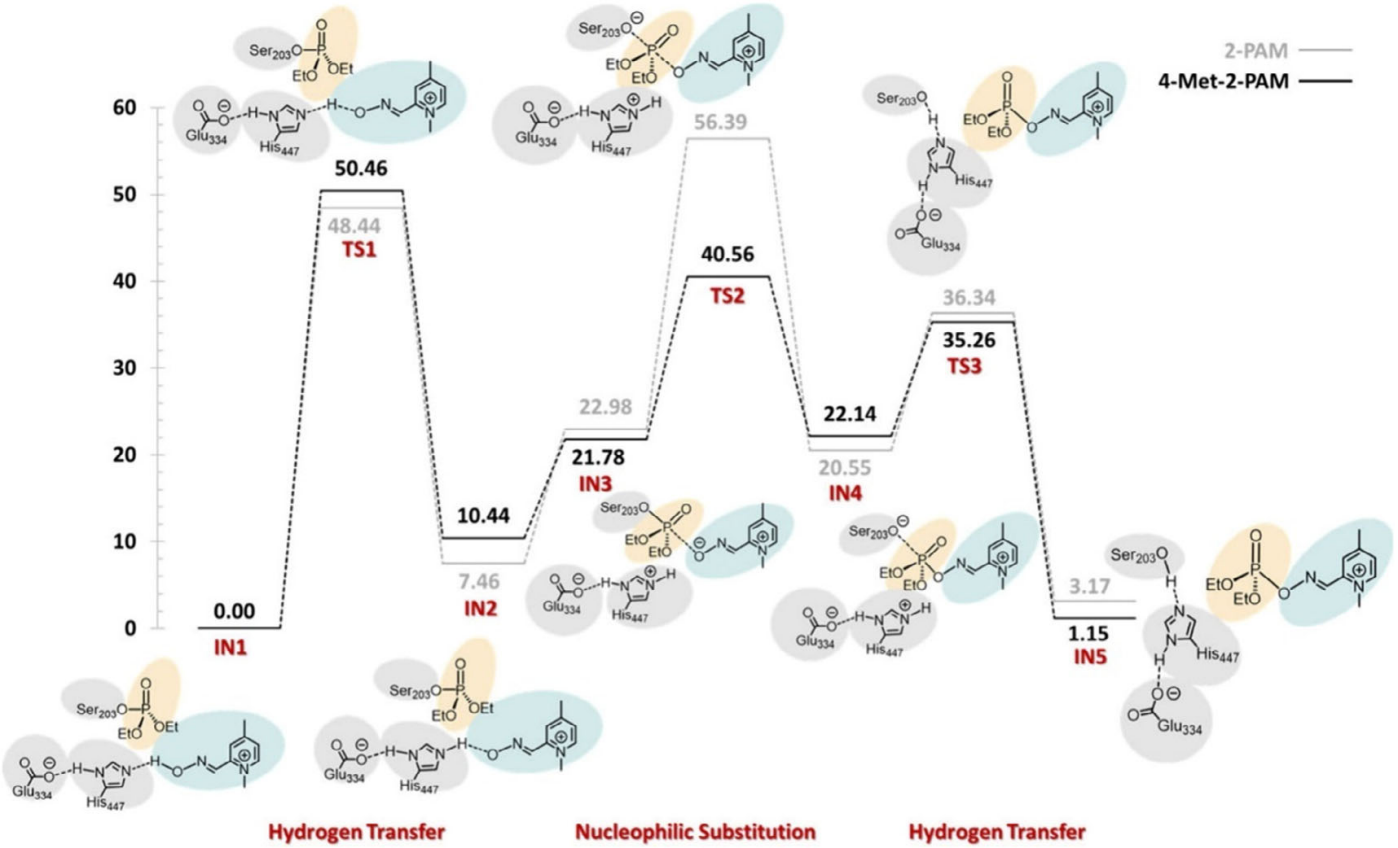
Solvent‐corrected relative energy profile for the reactivation of acetylcholinesterase (AChE) by 4‐Met‐2‐PAM (black line) and 2‐PAM (gray line), illustrating the hydrogen transfer, nucleophilic substitution, and final hydrogen transfer leading to the formation of free Ser203. Reproduced from ref [38]. Licensed under CC‐BY 4.0.

Building on these efforts, a recent contribution by Naweephattana et al. [38] offers a detailed mechanistic analysis of 2‐PAM and its methyl‐substituted analogs through QM/MM simulations. By examining paraoxon‐inhibited acetylcholinesterase (AChE), the study demonstrates that methyl substituents at the 4‐ and 6‐positions increase the negative charge density on the oxime oxygen, thereby enhancing nucleophilicity and lowering the energy barriers (Figure 4). The incorporation of dual methyl groups in 4,6‐dimethyl‐2‐PAM facilitates a more efficient nucleophilic attack and shifts the rate‐determining step from the substitution event to the preceding hydrogen transfer. This observation reveals how subtle steric and electronic modifications can reshape the energy profile of the reactivation process.

To conclude this part of the review, it is clear that the accumulated insights from recent computational studies delineate a coherent and evolving trajectory in the field of oxime‐mediated AChE reactivation. These advances, from Um's and Wang's articles, have progressively deepened our mechanistic understanding of oxime reactivity. The field has transitioned from early experimental observations, such as linear free energy relationships (LFERs) and resonance effects, through static DFT‐based energy profiles, to dynamic ab initio molecular dynamics (AIMD) simulations that capture free‐energy landscapes in real time. This evolution has led to a multifactorial paradigm in which oxime‐mediated detoxification is governed by a combination of electronic tuning (e.g., pK_a_ modulation and α‐effect enhancement), steric and orientational compatibility within the AChE gorge, solvation dynamics and proton transfer networks, and the transient accessibility of pentacoordinate phosphorus intermediates. This robust theoretical foundation now underpins the structure–reactivity relationships and rational design strategies that will be explored below.

## 3 Structure–Reactivity Relationships in Oxime‐Based Reactivators

In the previous section of this paper we have discussed, from a theoretical and computational point of view, the relevance of the structure of oximes on their reactivity with OPs. It should be noted that the standard treatment for OP poisoning has not changed significantly. Despite decades of research, it remains based on atropine and oximes as cholinesterase reactivators. The oximes used conventionally are still those established in the mid‐20th century, such as 2‐PAM and the classic oximes shown in Scheme 3 [39]. The main difficulty in the effectiveness of these oximes lies in their poor penetration into the central nervous system. Advancing the design of new oximes capable of overcoming this barrier is therefore a pressing need [19].

The design of effective oximes has traditionally centered on systematic modifications to their molecular scaffolds [40] By scaffold, we refer to the central architecture of the oxime (monopyridinium, bispyridinium, etc.) upon which structural adjustments are made to modulate activity. Early efforts focused on charged mono‐ and bis‐pyridinium compounds, but recent years have seen the field expand to include uncharged oximes and even nonoxime reactivators. This convergence between therapeutic urgency and molecular design has driven a wave of research focused on improving reactivity, selectivity, and bioavailability, laying the groundwork for structure–reactivity relationships and rational design strategies explored in this part of the review.

The earliest systematic comparisons of scaffolds focused on the structural dichotomy between monopyridinium oximes (analogs of monoquaternary pralidoxime) and bispyridinium oximes (such as obidoxime, HI‐6, etc.). Although both classes share the cationic pyridinium ring and the oxime moiety as the reactive α‐nucleophile, differences in geometry, electronic properties, and conformational flexibility have proven decisive for their nucleophilic performance at the phosphylated acetylcholinesterase (AChE) active site [40]. From a chemical perspective, monopyridinium oximes exhibit higher lipophilicity and lower polarity, which allows for somewhat improved tissue penetration. However, their limited binding affinity within the AChE gorge and the suboptimal orientation of the oxime group often compromise reactivation efficiency [41]. In contrast, bispyridinium scaffolds, despite being more polar and less permeable to the brain, benefit from preorganized dicationic structures that anchor more effectively within the enzyme's active site [42].

Despite extensive efforts, this scaffold‐based strategy has revealed inherent limitations. Minor structural changes can significantly alter reactivity profiles across different OP compounds, yet no single scaffold has demonstrated universal efficacy [43]. Oximes effective against sarin or VX often fail against soman or tabun, and efforts to improve blood–brain barrier penetration frequently compromise solubility or nucleophilic strength. These trade‐offs illustrate the narrow margin for chemical optimization and help explain why, despite the synthesis of hundreds of analogs, only a few have progressed to advanced pharmacological testing.

Mercey et al. reviewed structural modifications to oximes, including fluorination, sugar conjugation, and the use of prodrugs such as Pro‐2‐PAM [40]. They also examined nanoparticle‐based delivery systems, such as liposomes, mesoporous silica nanoparticles (MSNs), and human serum albumin carriers, as promising tools to overcome BBB limitations. In a critical clinical perspective, Worek, Thiermann, and Wille questioned the therapeutic relevance of oximes in human pesticide poisoning, emphasizing the lack of conclusive evidence and the absence of any experimental reactivator with a suitable clinical profile [43].

Complementing these structural and clinical evaluations, Costanzi et al. provided a comprehensive overview of the molecular mechanisms underlying nerve agent toxicity and the shortcomings of current treatments [44]. Their review highlighted ongoing efforts to develop nonoxime reactivators capable of reversing aged enzyme inhibition, as well as alternative therapeutic strategies including nicotinic antagonists and catalytic bioscavengers. Prchalova et al. [45] in another interesting review provides an overview and critical analysis of the strategies used to improve the bioavailability of oxime reactivators in the central nervous system. In this case, various approaches were discussed to overcome this limitation and improve the bioavailability of oxime reactivators in the CNS, including structural modifications. Their review covered liposomes modified with aptamers, solid lipid nanoparticles (SLNs), and metal–organic frameworks (MOFs), evaluating their performance in preclinical models.

If we look at published works that study the synthesis of oximes from a more mechanistic perspective, we must highlight the contributions of Radić et al. [46, 47, 48]. This team has systematically explored scaffold design through the synthesis and structural evaluation of extensive oxime libraries, ranging from conventional bispyridinium frameworks to neutral or partially ionizable hybrids. Their approach combines synthetic chemistry, crystallography, and enzyme kinetics to dissect how variations in linker length, substitution pattern, or charge distribution affect the positioning of the oxime group within the AChE active site and the resulting reactivation efficiency. The initial phase of this program involved a structure–activity analysis of 134 novel compounds, most of which remain largely neutral at physiological pH. The study primarily focused on imidazole aldoximes and N‐substituted 2‐hydroxyiminoacetamides [48]. The authors report the synthesis and biological evaluation of a new series of cholinesterase (ChE) oxime reactivators. Their reactivation capabilities were assessed by comparing relative reactivation rate constants with those of 2‐pyridinealdoxime, targeting four organophosphate conjugates (sarin, cyclosarin, VX, and parathion) bound to human acetylcholinesterase (hAChE). The ten most effective oximes, predominantly hydroxyiminoacetamide derivatives (for hAChE) and imidazole‐containing aldoximes, also exhibited appreciable activity against tabun‐conjugated ChE. Reactivation kinetics were analyzed in terms of apparent affinity and maximal reactivation rate. The pH‐dependent differences in reactivation point to multiple ionization states of oximes, yet the decisive factor for efficient ChE reactivation appears to be their structural access to the phosphonyl or phosphoryl phosphorus atom within the narrow confines of the enzyme gorge.

**FIGURE 5:**
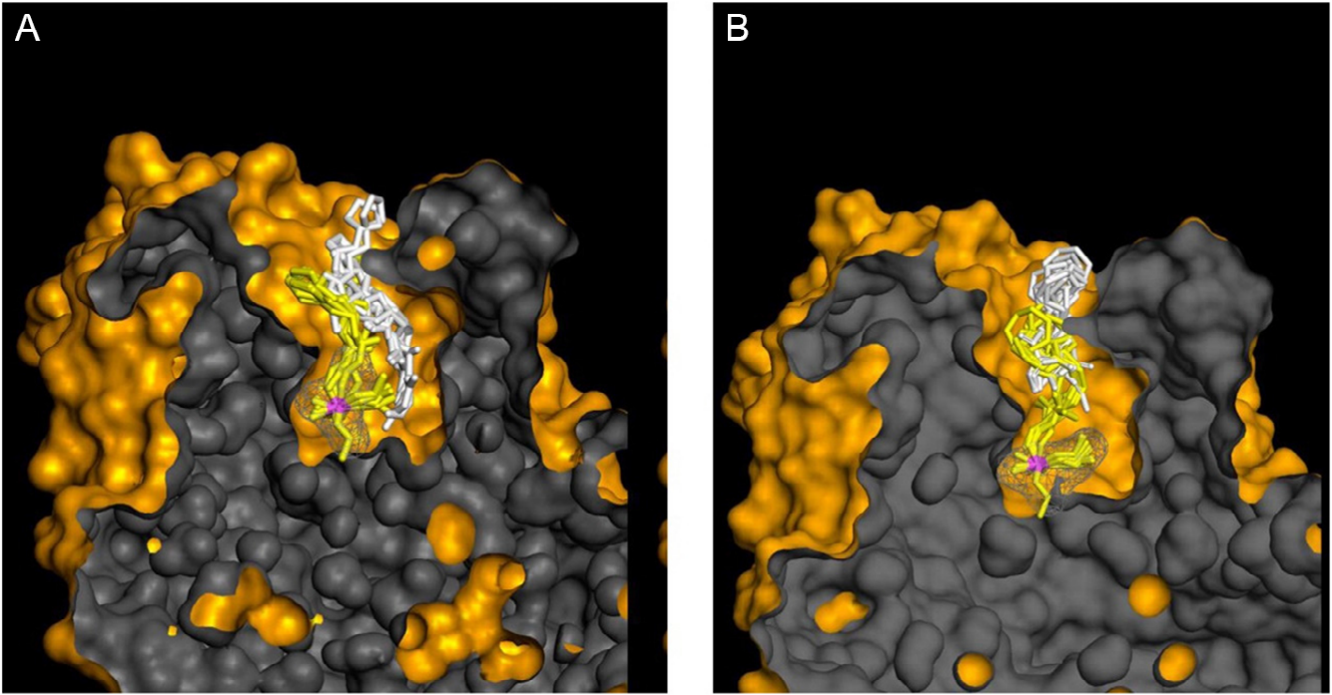
Computational molecular modeling of VX‐inhibited AchE showing the reversible Michaelis type complex (white sticks) and covalent pentacoordinate trigonal bipyramidal intermediate (yellow sticks) for interaction of initial lead oxime RS41A (A) and the lead oxime RS194B (B). Ten conformers of each oxime are shown in each of two interaction states. The phosphorus atom is colored purple. The solvent‐accessible part of the hAChE Connolly surface is represented in orange, and the solvent‐inaccessible part of the hAChE molecule is in dark gray. Pronounced overlapping similarity in global geometries of the reversible complex (white sticks) and trigonal bipyramidal intermediate (yellow sticks) was observed for RS194B oxime, but not with RS41A oxime. Reproduced from ref [46]. Licensed under CC‐BY 4.0.

In this line of work and using RS41A, the oxime with the best performance in the previous article, as a starting point, a series of systematic structural modifications was undertaken (Scheme 7) [46]. This led to a comprehensive characterization of a novel series of N‐substituted 2‐hydroxyiminoacetamido alkylamine reactivators targeting nerve agent‐conjugated human acetylcholinesterase (hAChE). Through iterative optimization, RS41A was refined into the more effective reactivator RS194B. Computational modeling of two key reaction steps consistently revealed that RS194B exhibits a greater geometric similarity between the reversible complex and the trigonal bipyramidal intermediate, in contrast to RS41A (Figure 5). The outstanding intrinsic reactivation potency of RS194B, partly attributed to its favorable interaction with the peripheral site of AChE, combined with its low toxicity, positions it as a promising lead for a broad‐spectrum antidote. This compound demonstrated efficacy in OP‐exposed mice, both in post‐exposure treatment and in combined prophylactic‐antidotal regimens.

A more recent and conceptually innovative contribution came with the introduction of neutral or partially ionizable bis‐oximes designed to cross theBBB in their uncharged form and to reactivate in ionized form once bound to AchE [47]. Starting from X‐ray structures of the CNS‐penetrant monoxime RS194B bound reversibly to both native and VX‐conjugated hAChE, researchers designed seven uncharged acetamido bis‐oximes as potential antidotes. In these molecules, both oxime groups are connected to a common saturated heterocyclic core. Variable protonation of the heterocyclic amines and oxime moieties generated an equilibrium among up to 16 ionization states, including uncharged species capable of crossing the BBB and zwitterionic forms optimized for reactivation. Structural analysis confirmed that in one bis‐oxime, LG‐703, reorientation of the oxime group positioned it within the active site, facilitating reactivation. Experimental data revealed detailed structure–activity relationships for several CNS‐targeted, uncharged aliphatic bis‐oximes, which behave as protonation‐dependent, conformationally adaptive “smart” antidotes. Notably, these compounds retain a higher proportion of uncharged species at physiological pH (7.4) than RS194B, suggesting improved BBB permeability and CNS accessibility.

A particularly compelling example of scaffold‐driven chemical design is provided by Saint‐André et al. [49], who synthesized and systematically evaluated a diverse library of α‐nucleophiles, including aryl‐ and pyridyl‐aldoximes, amidoximes, and hydroxamic acid derivatives. Using phenyl phosphonothioate (PhX) as a safe surrogate for VX, their screening strategy combined HPLC‐based kinetic monitoring with LC‐MS product identification, enabling both quantitative analysis of substrate consumption and mechanistic insight into the hydrolysis process. Unlike previous studies, the researchers adopted a mechanistically oriented approach to explore structure–activity relationships among these nucleophiles. The new oximes and amidoximes studied, uncharged nucleophiles, show reactivity comparable to or greater than that of 2‐PAM. In the specific case of benzaldoximes, those with a hydroxyl group or a carboxyl group in the ortho position showed enhanced activity towards hydrolysis of PhX as compared to the unsubstituted oxime. This reactivity was explained by the intramolecular hydrogen bond between the hydroxyl group and the nitrogen atom of the oxime. It is suggested that a lower pK_a_ results in an increase in oxime concentration, leading to the higher hydrolytic activity observed. Among all the oximes studied, the hydrolysis reactivity of 3‐hydroxypyridyl‐amidoxime stands out, surpassing that of 2‐PAM oxime; however, due to the formation of isoxazole derivatives, this compound shows no catalytic activity.

Kallol K. Ghosh has developed much of his research using colloidal systems as organized media for studying the kinetics of organophosphate (OP) decontamination. A complementary line of research led by Ghosh and coworkers has focused on the design, synthesis, and mechanistic evaluation of new pyridinium oximes. Across a series of studies published the group systematically explored structure–reactivity relationships among mono‐ and bis‐pyridinium oximes using in vitro kinetic assays with Electrophorus electricus AchE inhibited by organophosphorus compounds. In addition to its own research, a review conducted in 2014 by this research group delves into structural modifications of AchE reactivators, highlighting strategies designed to improve both their binding affinity and their ability to penetrate the blood–brain barrier [50].

In 2010, Ghosh contributed to a collaborative in vitro study that focused on the biochemical evaluation of a new oxime. In one such study, a new bis‐quaternary oxime, K027 (Scheme 7), was synthesized and tested for its ability to reactivate AchE inhibited by a broad range of organophosphates: sarin, cyclosarin, tabun, VX, Russian VX, soman, methylchlorpyrifos, paraoxon, and DDVP [51]. K027 was able to restore AchE activity to over 10% for most inhibitors, a level considered sufficient to prevent fatal outcomes in intoxicated organisms. However, its efficacy was limited against cyclosarin‐ and soman‐inhibited AchE.

**SCHEME 8:**
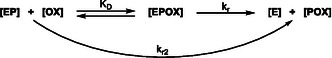
Reaction mechanism. Scheme created by the author based on ref [52].

In a comprehensive kinetic study, Ghosh's group evaluated ten structurally diverse pyridinium oxime‐based reactivators for their in vitro efficacy in reactivating electric eel AchE inhibited by DFP and paraoxon [52]. The oximes used were mono‐quaternary and bis‐quaternary (symmetrical and asymmetrical). The kinetic rate constants observed in the study highlight the importance of both the oxime group's position and the nature of the connecting linker in enhancing reactivation efficacy. Using a Michaelis–Menten‐based formalism, the kinetic parameters were quantitatively determined (Scheme 8). The results showed that the reactivation rate constants are strongly influenced by the chemical structures of both oximes and inhibitors. Although the reaction rate (*k*_r_) remained relatively stable, the dissociation constant (*K*_D_) varied considerably. Since specific oxime reactivity (*k*_r2_) depends on both intrinsic chemical activity and binding affinity, the parameter showed substantial variation across the compounds tested. These findings reinforce the challenge of designing a universal oxime‐based reactivator effective against all organophosphates. Oximes with aliphatic linkers outperform those with unsaturated or aromatic linkers in reactivation efficiency. For paraoxon‐ and DFP‐inhibited AchE, the optimal linker length between pyridinium rings is three to four carbon atoms, offering moderate but effective reactivation.

Ghosh's group also conducted a kinetic study evaluating a series of xylene‐linked carbamoyl bis‐pyridinium mono‐oximes as reactivators of AchE inhibited by various organophosphates, including paraoxon, DFP, sarin, and VX [53]. The oximes differed in linker position and functional group placement. Reactivation efficiency was assessed via kinetic parameters and compared to conventional oximes (obidoxime, 2‐PAM and TMB4). The study highlights the influence of molecular architecture on oxime performance and the challenge of designing a universal reactivator effective across structurally diverse OPs. Based on the previous kinetic study, Ghosh's group conducted a comparative in vitro investigation into the physicochemical properties and reactivation efficiency of structurally diverse oximes against paraoxon‐inhibited AchE [54]. This work assessed key molecular descriptors (including pK_a_, logP, and polar surface area) and revealed that oximes with aliphatic linkers outperformed xylene‐linked analogs in reactivation potency, while also offering insights into their potential for CNS penetration. The study highlights the need to balance nucleophilic potency with physicochemical traits to design more effective antidotes.

Building on these efforts, Ghosh's group expanded their scope beyond pyridinium‐based oximes to explore alternative scaffolds with improved physicochemical profiles. In a 2016 study, they presented a series of bis‐ and mono‐quaternary imidazolium aldoximes and evaluated their in vitro reactivation potential against VX‐ and sarin‐inhibited human AchE [55]. It was also studied the effect of absence and presence of pyridinium ring along with imidazole ring. Among the tested compounds, one of the bis‐quaternary oximes in particular showed reactivation efficacy comparable to 2‐PAM and obidoxime, particularly against sarin. The study emphasized the role of molecular descriptors such as pK_a_, logP, and polar surface area in predicting reactivation potency and blood–brain barrier permeability. In another article, butene‐linked bis‐pyridinium mono oximes were tested in vitro against AchE inhibited by various nerve agents and pesticides. The results, consistent with previous work, showed improvements over known oximes, reinforcing the trend that small structural changes can yield slightly enhanced reactivation efficacy [56].

Continuing this line of inquiry, a 2020 publication introduced glycosylated imidazolium aldoximes designed to exploit glucose transport mechanisms for enhanced CNS penetration [57]. These sugar‐conjugated oximes were tested against paraoxon‐ethyl and paraoxon‐methyl inhibited electric eel AchE. Although these compounds did not outperform standard oximes in terms of reactivation efficacy, they exhibited favorable lipophilicity and physicochemical properties, indicating potential for further optimization. Collectively, these studies reflect a strategic shift toward designing oximes with enhanced CNS bioavailability, balancing nucleophilic potency with molecular architecture to overcome the limitations of current antidotes.

**SCHEME 9:**
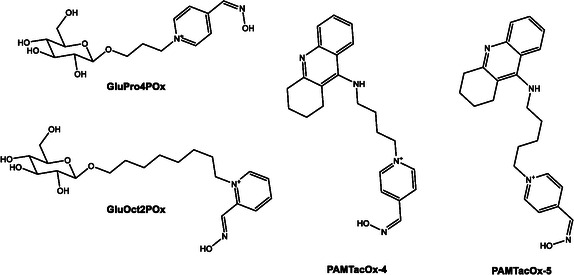
Functionalized oxime derivatives incorporating carbohydrate or tacrine moieties for improved antidotal performance.

A novel class of sugar–oxime conjugates designed to penetrate theBBB via glucose transporter‐mediated uptake was introduced previously by Garcia et al. [58] Fourteen sugar–oximes were synthesized and tested for reactivation of human AchE and butyrylcholinesterase (BchE) inhibited by various OPs, including VX and paraoxon. The most promising compound, GluOct2Pox (Scheme 9), showed reactivation potency comparable to 2‐PAM for AchE, while GluPro4Pox (Scheme 9) demonstrated low toxicity and good BchE reactivation. Toxicological studies in mice and guinea pigs confirmed the safety profile of GluPro4Pox (Scheme 9), with no observable histopathological damage. This work laid the foundation for CNS‐targeted oxime delivery using sugar‐based carriers, highlighting the potential of glycosylated systems for systemic and central detoxification.

Recent contributions by Choi and co‐workers have significantly advanced the rational design of hydrophilic α‐nucleophiles as catalytic scavengers of organophosphates (OPs). In two complementary studies, they constructed libraries of oximes based either on pyridinium aldoxime cores [59] or on 2,3‐butanedione onoxime (diacetyl onoxime, DAM) and monoisonitrosoacetone (MINA) cores tethered to polar scaffolds [60], systematically modulating molecular size, hydrophilicity, and acidity to overcome the limitations of Dekon‐139. Dekon‐139, the potassium salt of 2,3‐butanedione onoxime, is the active ingredient in the FDA‐approved lotion for skin decontamination against OP agents. However, its neutral form is rapidly absorbed through the skin, which can cause adverse systemic effects. Among the various experimental techniques, the results obtained through high‐performance calorimetric tests with paraoxon (POX) as a model OP stand out. The kinetics of POX inactivation by these compounds under various temperature and pH conditions were determined in this way. The negatively charged oximate form was shown to be at least 10 times more reactive than the neutral oxime. Additionally, the inactivation of the organophosphate proceeds through an endothermic process. Mechanistic investigations using 1H‐NMR spectroscopy and UV–Vis kinetics provided direct evidence for catalytic hydrolysis of POX with simultaneous 4‐nitrophenol release. Ex vivo porcine skin assays confirmed the superior decontamination efficacy of several lead compounds over Dekon‐139, with reduced POX penetration and lower residual levels in skin and receptor compartments. The MINA series exhibited more efficient POX inactivation than the DAM series, following a clear structure–reactivity trend. This enhanced performance is attributed to the favorable physicochemical properties of the MINA compounds, including greater oxime acidity and reduced steric hindrance, both contributing to increased nucleophilicity. Additionally, six lead compounds were identified with two‐ to three‐fold higher reactivity compared to Dekon‐139

**FIGURE 6:**
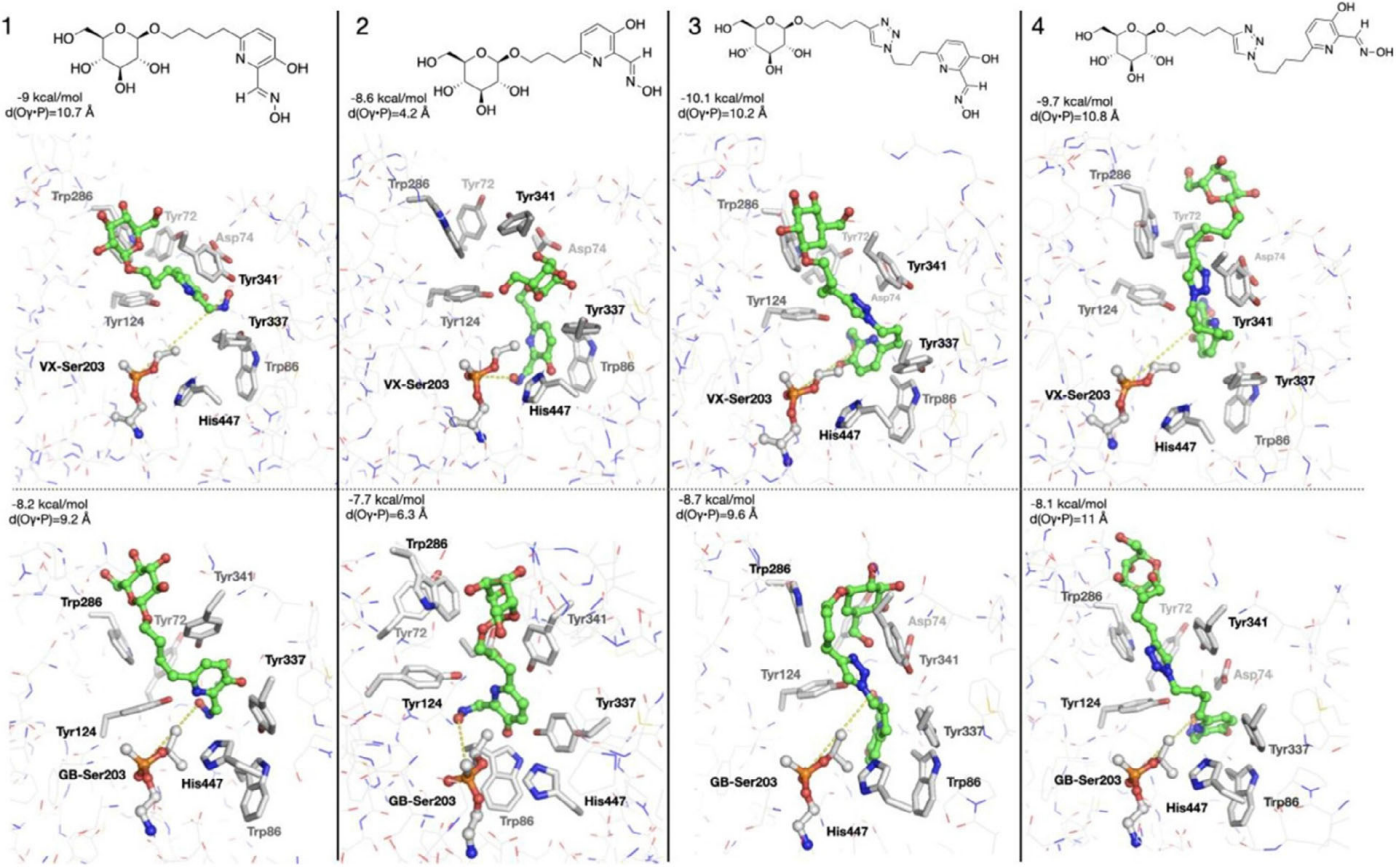
Molecular docking of selected oximes in the active site of VX and GB‐inhibited human acetylcholinesterase (respectively top and bottom panel). The binding energy determined by the scoring function of Autodock Vina and the distance between the phosphorus atom of VX or GB and the oxime oxygen atom are indicated in the top left corner of each docking pose. Reproduced from ref [61], with permission from American Chemical Society. Copyright 2017.

Koning et al. [61] introduced a modular scaffold‐based strategy to enhance oxime reactivity and central nervous system (CNS) penetration. Using the Ugi multicomponent reaction, they synthesized a small library of hybrid compounds in which an oxime nucleophile was covalently linked to a peripheral site ligand via a flexible spacer. This design enabled fine‐tuning of both affinity and orientation within the AChE active site, as confirmed by docking simulations and kinetic analyses against sarin‐, cyclosarin‐, and tabun‐inhibited enzymes (Figure 6). Notably, several imidazolium‐based derivatives outperformed 2‐PAM in vivo, achieving superior brain reactivation and seizure suppression in sarin‐exposed rats. These findings reinforce the potential of scaffold engineering not only to optimize nucleophile positioning for SN2 displacement, but also to modulate pharmacokinetic properties critical for CNS efficacy.

In a complementary study with a more straightforward synthetic approach, Gambino et al. [62] applied a methyl scan to the pyridinium ring of 2‐PAM to systematically evaluate steric and electronic effects on reactivation potency. Structure–activity relationship (SAR) analysis revealed that methylation at positions 4 and 6 enhances reactivity, primarily by promoting hydrophobic interactions and improving the spatial orientation of the reactive groups within the AChE gorge. In addition to favoring these optimal conformations, methylation also contributed to improved permeability across the blood–brain barrier.

Ongoing experimental efforts have focused on how structural modifications in oximes influence their therapeutic potential. In this context, Kim et al. [63] introduced a series of tacrine–pyridine hybrid oximes featuring variable linker lengths. This design aimed to exploit dual‐site interactions within acetylcholinesterase (AChE), engaging both the catalytic and peripheral sites. Compounds with intermediate linker lengths, notably PAMTacOx‐4 and PAM‐TAcOx‐5 (Scheme 9) showed enhanced reactivation of paraoxon‐inhibited AChE compared to standard oximes like 2‐PAM and HI‐6, supported by molecular docking simulations that confirmed favorable positioning of the oximate group for nucleophilic attack.

In a complementary approach, Zorbaz et al. [37] synthesized chlorinated bispyridinium mono‐oximes derived from known reactivators such as K027 (Scheme 3). The introduction of ortho‐positioned chlorine atoms lowered the pK_a_ of the oxime group, increasing oximate formation at physiological pH and improving reactivation efficiency against sarin‐, VX‐, and cyclosarin‐inhibited human AChE. The most potent compound, DiClProp2Pox (Scheme 7), also demonstrated improved blood–brain barrier permeability in PAMPA assays (parallel artificial membrane permeation assays) and effective ex vivo degradation of nerve agents in human whole blood, highlighting its therapeutic promise.

More recently, Knittelova et al. [64] developed halogenated monoquaternary oximes based on the 2‐PAM scaffold, focusing on C3 and C5 substitution patterns. These modifications led to improved nucleophilicity, reduced pK_a_ values, and enhanced reactivation of AChE and BChE inhibited by various organophosphate surrogates. The C3‐fluorinated analog emerged as particularly effective, outperforming 2‐PAM in both reactivation screening and kinetic assays. The results obtained in this article suggest, once again, that steric effects or specific interactions with enzyme residues could affect reactivation capacity and should be studied further in research into broad‐spectrum cholinesterase reactivators.

This part of the review highlights how scaffold‐centered strategies demonstrate the versatility of structural chemistry in modulating oxime reactivity. The broad range of scaffolds explored has enabled systematic investigation of key chemical parameters, such as polarity, pK_a_, and spatial orientation. Initial efforts focused on modest modifications to bispyridinium derivatives, but more recent studies have introduced libraries of hydrophilic α‐nucleophiles and hybrid architectures. These developments have significantly improved in vitro performance. However, translating these compounds into clinically effective antidotes remains a challenge, as pharmacokinetic limitations and poor blood–brain barrier penetration persist.

An important line of research focuses not only on improving the structure of oximes, but also on designing molecular environments that favor nucleophilic activity. Incorporating oximes into colloidal media, nanoparticles, or supramolecular assemblies allows for control over microreactivity and enhancement of catalytic performance, ultimately leading to new therapeutic alternatives for the reactivation of inhibited acetylcholinesterase (AChE).

## 4 Micellar Systems as Reactive Microenvironments

The transition from conventional solution‐phase studies to self‐assembled and nanostructured systems marks a natural evolution in the effort to enhance oxime reactivity. Despite the wide range of possible structures, colloidal media share a common feature: they influence both the solvation equilibrium and the transition state. As a result, these systems offer new opportunities beyond what has been kinetically characterized in conventional aqueous environments.

**SCHEME 10:**
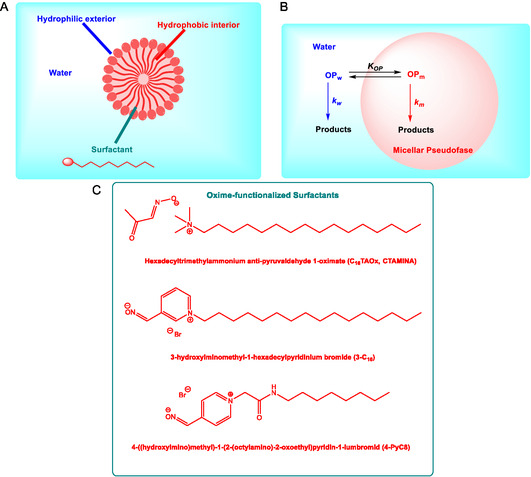
Graphical representation of the structure of a micelle (A), simple pseudophase model (B), and the structure of oxime‐functionalized surfactants CTMINA, 3C16, and 4‐PyC8 (C).

Micellar effects represent one of the most extensively studied examples of how surfactant‐based systems can influence the kinetics of organic reactions in aqueous media. These colloidal assemblies offer powerful means to control reaction rates and equilibrium by altering the local environment of the reactants. Acceleration in micellar solutions is typically attributed to two key factors: (1) the concentration of reactants within the micelle pseudophase and (2) the distinct microenvironment provided by the surfactant interface, which can significantly enhance reactivity compared to bulk aqueous solution (Scheme 10) [65]. The systematic study of reaction kinetics in colloidal media began in the 1970s, with foundational contributions from F. M. Menger and C. A. Bunton [66, 67]. Menger's early work on bile salt micelles provided one of the first quantitative analyses of micellar catalysis, including the use of oxime‐functionalized micelles to mimic enzymatic activity [68]. Concurrently, Bunton conducted a series of seminal investigations demonstrating the capacity of micelles to act as organized reaction media, effectively modulating nucleophilic substitution and hydrolysis reactions through microenvironmental effects, including substrate solubilization, charge stabilization, and transition state organization [69, 70, 71, 72, 73, 74, 75].

A parallel line of research in colloidal systems can be traced through the contributions of Buncel, whose work significantly advanced the mechanistic understanding of micellar reactivity.

Alongside Bunton's investigations, Buncel and Toullec explored reactive counterion micelles incorporating oximate ions as nucleophilic partners, shedding light on the nuanced interplay between micellar structure and nucleophilic behavior. Their studies demonstrated that replacing inert halide counterions with nucleophilic species such as anti‐pyruvaldehyde oximate dramatically enhanced the hydrolysis rates of phosphate triesters, including paraoxon and fenitrothion [76, 77]. Toullec and Couderc investigated the hydrolysis of phosphate triesters (including paraoxon) using micelles formed by hexadecyltrimethylammonium anti‐pyruvaldehyde 1‐oximate (C_16_TAOx) [76]. These micelles incorporate oximate counterions, which act as α‐effect supernucleophiles. The system achieved rapid hydrolysis of paraoxon (half‐life ≈ 2 min) under mild pH conditions (~8), making it suitable for detoxification applications. The authors analyzed the kinetics using the pseudophase ion‐exchange (PIE) model, but observed deviations that suggested additional effects, possibly linked to changes in interfacial potential and ionic strength.

**SCHEME 11:**
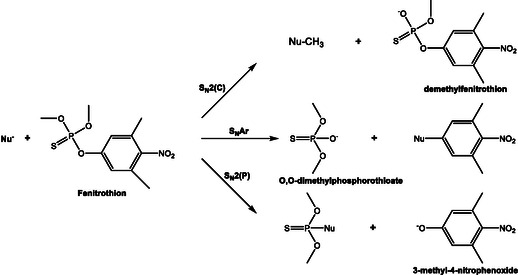
Reaction mechanism. Scheme created by the author based on ref [77].

Buncel, Toullec, and coworkers extended this approach to the fenitrothion, comparing its degradation in micellar systems containing either hydroxide (CTAOH) or oximate (CTAMINA) counterions [77]. It was demonstrated that cationic micelles with reactive counterions not only accelerate hydrolysis but also modulate regioselectivity, revealing dual S_*N*_2(P) and S_*N*_2(C) pathways and allowing inference of substrate orientation within the micelle (Scheme 11). Using ^31^P‐NMR and UV–vis spectroscopy, the authors proposed an orientation of the substrate within the micelle: the *P* = S moiety was exposed to the aqueous pseudophase, while the aromatic and alkyl groups were buried in the micellar core. This orientation favored attack at phosphorus and highlighted the utility of oximate‐functionalized micelles for environmentally relevant decontamination strategies.

These studies by Buncel and collaborators, conducted in parallel with those of Toullec and during the same period, reinforce and extend the mechanistic understanding of micellar catalysis in the degradation of OPs. They established that oxygen nucleophiles, including α‐nucleophiles like hydroperoxide and oximates, react with fenitrothion via a concerted mechanism, supported by Brønsted‐type correlations and significant α‐effects, with no evidence for metaphosphate intermediates [78]. Their 2005 work extended the methodology to soil matrices, showing that reactive counterion micelles, especially those incorporating α‐nucleophiles like CTAOOH, achieve rapid degradation of fenitrothion even in complex environmental solids [79]. They conducted also a systematic study of CTA‐oximates with varying pK_a_ and lipophilicity, dissecting the kinetic contributions of S_*N*_2(C) and S_*N*_2(P) pathways using the PPIE model, and revealing a decreasing α‐effect with increasing nucleophile basicity [80]. Finally, in 2007, they explored the influence of surfactant chain length and ionic strength on alkaline hydrolysis, showing that longer chains enhance substrate binding and rate acceleration, while high bromide concentrations induce micellar shape transitions that challenge the applicability of the PPIE model [81].

These studies build upon Bunton's early work on micellar catalysis, helping to clarify the mechanistic basis for oximate‐mediated dephosphylation in confined colloidal systems. During those same years, Bunton also collaborated with Simanenko's group on the development of oxime‐functionalized imidazolium surfactants, which formed reactive micelles capable of promoting nucleophilic attack at phosphoryl and sulfonyl centers [82, 83]. These micellar systems dramatically accelerate S_*N*_2‐type cleavage of sulfonate, phosphate, and phosphonate esters up to 12 000‐fold compared to monomeric analogs, primarily due to the high local concentration of the oximate group within the micelle interface. While the intrinsic nucleophilicity of the oxime remains largely unchanged, the micellar environment enhances reactivity by concentrating both substrate and nucleophile in a confined pseudophase.

Along the same lines of research, the Litvinenko Institute of Physical Organic Chemistry group has continued working on these colloidal systems. A comprehensive series of studies has established a robust experimental framework for micellar and colloidal systems designed to hydrolyze organophosphorus (OP) compounds [84, 85, 86, 87, 88, 89, 90, 91].

**FIGURE 7:**
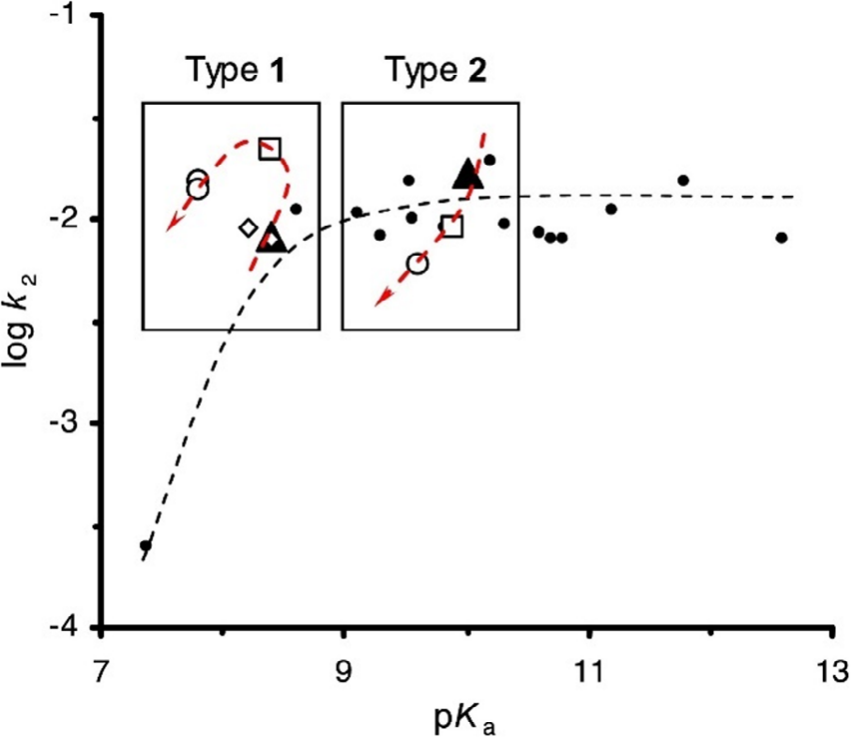
Bronsted plot for the reaction of oximate ions with NPDEP in water, zwitterionic micelles of oxime‐functionalized surfactants, and their mixed micellar systems with conventional cationic CTABr and nonionic Tween 80 surfactants. Reproduced from ref [89], with permission from Elsevier. Copyright 2017.

Spanning over a decade, their work systematically explored oximate‐functionalized surfactants, gemini micelles, and ionic liquid (IL)‐derived amphiphiles as supernucleophilic media. Using Brønsted‐type correlations (Figure 7) and pseudophase partitioning models, they quantified how micellar charge, surfactant architecture, and substrate hydrophobicity influence reaction rates, often achieving 10^2^–10^3^‐fold accelerations relative to aqueous conditions. In later contributions, the group introduced IL‐based surfactants incorporating amino acid fragments, such as phenylalanine, to enhance biodegradability and environmental compatibility [90]. These amphiphilic oximes demonstrated rapid cleavage of OP simulants under mild pH, with half‐lives under 1 minute, and exhibited measurable shifts in apparent pK_a_ depending on micellar composition. While mechanistic depth remains limited, focused primarily on kinetic trends and empirical modeling, the collective body of work provides a valuable quantitative basis for designing tunable, green micellar systems for OP detoxification.

Ghosh's research group is arguably the most prolific in this field, having published a substantial number of studies. Among the various strategies they have investigated, micellar systems incorporating α‐nucleophiles—such as oximes and hydroxamates—have emerged as particularly promising. These systems leverage microenvironmental effects and structural fine‐tuning to significantly enhance nucleophilic reactivity [92, 93, 94]. Continuing their long‐standing interest in micellar catalysis, Ghosh and coworkers systematically investigated oxime‐functionalized pyridinium surfactants as nucleophilic micellar catalysts. Initial studies focused on model esters, establishing how micellar charge, alkyl chain length, and headgroup structure influence catalytic efficiency [95, 96]. In a follow‐up study [96], the researchers replaced model compounds with actual organophosphorus agents and prepared mixed micelles combining oxime‐functionalized and conventional surfactants. As expected, surfactants with longer alkyl chains and aromatic head groups exhibited lower critical micelle concentrations (CMC). In addition to reduced CMC values, the catalytic efficiency of the system also improved. The optimal system (3‐C_16_/CPB) achieved rapid OP degradation under mild conditions, demonstrating the potential of micellar oxime systems for environmental decontamination. Surprisingly, this study does not apply the pseudophase model to fit the experimental velocity constants and extract the standard kinetic parameters—a methodology commonly employed in most of this group's previous work. Nonetheless, it provides valuable insights for the development of effective systems aimed at degrading toxic pesticides and nerve agents.

**SCHEME 12:**
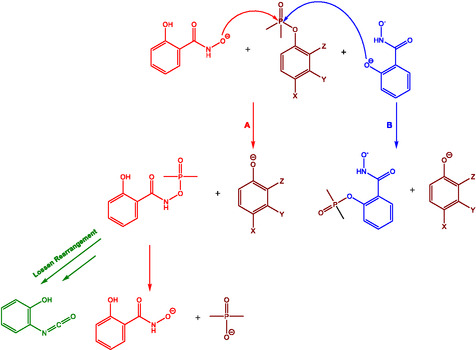
Reaction mechanism. Scheme created by the author based on ref [98].

Hydroxamate ions, derived from hydroxamic acids, are potent α‐nucleophiles capable of cleaving carboxylate, phosphate, and sulfonate esters. Another article by this research group reviewed their own research progress on this topic in reactivity in micellar media, emphasizing the α‐effect, which enhances nucleophilicity beyond that predicted by basicity [97]. Particularly noteworthy is the discussion confirming the relevance of the Lossen rearrangement within micellar environments. This transformation involves the conversion of O‐activated hydroxamic acids into reactive isocyanate intermediates, which can subsequently yield ureas in the presence of amines or liberate amines upon hydrolysis. Ghosh's group further demonstrated the nucleophilic reactivity of hydroxamate ions in the cleavage of phosphate (BDNPP) and carboxylate (PNPB) esters, proposing a mechanism analogous to the Lossen rearrangement, facilitated by cationic CTAB micelles [98]. In agreement with the mechanistic picture proposed by Ghosh and coworkers, salicylhydroxamate reacts along two competitive pathways: path A, nucleophilic attack through the hydroxamate functionality at phosphorus, and path B, attack via the phenolate group (Scheme 12). Under these conditions, the O‐phosphorylated hydroxamate generated in the course of the reaction undergoes a Lossen‐type rearrangement to phenyl isocyanate, which is subsequently trapped (e.g., by benzohydroxamic acid) to give O‐phenylcarbamyl benzohydroxamate. The evolution of key species and products (DNP, DNPP, aromatic‐substituted products, and the carbamylated hydroxamate) was monitored by NMR and (ESI/EI)‐MS, supporting this assignment.

A comprehensive review by Ghosh and coworkers summarized the group's extensive contributions to the development of oxime‐functionalized surfactants and micellar systems for organophosphate detoxification, integrating the mechanistic, kinetic, and physicochemical findings accumulated up to 2015 [99]. This overview emphasized micellar rate enhancement, the α‐effect in oximate reactivity, and the design of functional surfactants, including pyridinium and imidazolium derivatives, as well as polymeric and metallomicellar systems.

Building upon the foundational work synthesized in the 2015 review, Ghosh and collaborators have significantly expanded the scope of micellar and organized media for organophosphate detoxification, incorporating new nucleophilic systems and reaction environments. In 2017, the research group conducted a detailed kinetic study on the hydrolysis of paraoxon, parathion, and fenitrothion using pyridine‐based oximate and hydroxamate ions in cationic micellar media (CTAB and TTAB) [100]. The study revealed that ortho‐substituted oximes (e.g., 2‐PyOx^‐^) exhibit superior nucleophilic reactivity compared to their para analogs, due to favorable electronic interactions. Hydroxamate ions, although less reactive than oximates, showed promising catalytic activity under micellar conditions. The authors applied the pseudophase model to quantify micelle–nucleophile interactions, demonstrating significant micellar rate enhancements. Notably, salicylaldoxime (SOx^‐^) acted as a bifunctional nucleophile, attacking both *P* = O and *P* = S centers, and exhibited the highest micellar association constants and catalytic efficiency.

In 2019, the group expanded beyond micellar systems by introducing a biosensor platform based on carbon quantum dots (CQDs) for dual‐mode detection of AChE inhibitors using fluorimetry and colorimetry [101]. The system enabled sensitive identification of both reversible (e.g., CPC, Triton X‐100) and irreversible (e.g., paraoxon and chlorpyrifos) inhibitors. Notably, it also allowed real‐time monitoring of AChE reactivation with monopyridinium oximes (2‐PAM, 3‐PAM, and 4‐PAM), showing efficiencies consistent with Ellman's assay. This work marked a shift toward nanomaterial‐assisted strategies for detection and detoxification, combining sensitivity, visual output, and biochemical relevance.

Finally, in collaboration with the Ukrainian group previously discussed, Ghosh coauthored a study on biodegradable ionic liquid‐derived amphiphilic oximes, which serves as a fitting conclusion to his contributions in this field [90]. These IL‐based surfactants, incorporating amino acid fragments such as phenylalanine, demonstrated rapid cleavage of organophosphate simulants under mild conditions and achieved measurable biodegradability in closed bottle tests. Under optimal concentration conditions, the oxymolysis of the organophosphate triester PNPDPP by 4‐PyC8 (an amphiphilic oxime featuring an octylamide tail linked to a pyridinium headgroup, Scheme 10) proceeds with half‐life times of approximately 62 s at pH 8.37 and 40 s at pH 9.00. This demonstrates that, through nucleophilic attack by an α‐effect oximate, the system achieves efficient cleavage of the model organophosphate within 1 minute under mild aqueous conditions. This work exemplifies the convergence of supernucleophilic design and environmental compatibility and reflects the broader trajectory of the field toward green and tunable micellar systems for organophosphate detoxification.

Other groups have explored the mechanistic foundations of micellar catalysis using surface‐active ionic liquids, notably the work by Bica et al. [102], which provides complementary insights into anion‐dependent reactivity in micellar media. This study examined how variations in the counterion of surface‐active ionic liquids (SAILs) affect their catalytic behavior in aqueous micellar systems. Using a series of 1‐dodecyl‐3‐methylimidazolium‐based ILs with halide and sulfonate anions, they showed that more hydrophobic or polarizable anions (e.g., I^‐^, OTs^‐^) promote micelle formation at lower concentrations and affect aggregate morphology. The study focused on the hydrolysis of PNPDPP with acetaldoxime, revealing that reaction rates peak near the CMC and correlate with the degree of anion binding to the micelle. Loosely bound anions like Cl^‐^ allow better access of the nucleophile to the interface, enhancing reactivity, while strongly bound anions compete more effectively, reducing catalytic efficiency. This work complements Ghosh's approach by showing how micellar reactivity can be modulated externally through ionic composition, offering a tunable platform for reaction control in water.

Overall, the cumulative research outlined above defines the competence and the built‐in shortcomings of micellar catalysis as an organophosphate detoxification platform. Classical surfactant aggregates, conventional or ionic liquid‐borne, have demonstrated remarkable rate enhancement and controllable reactivity by means of electrostatic preorganisation, microenvironmental polarity, and confining effects. The introduction of functionalized surfactants with oxime‐ or hydroxamate‐bearing headgroups and the systematic treatment with pseudophase kinetic models has given us a quantitative picture of remorseless detail regarding how nucleophilic reactivity is affected at the micellar interface. Nevertheless, in spite of their experimental versatility and mechanistic richness, micellar systems seem to be at the plateau in terms of their usefulness. Although still valuable model media in terms of examining α‐nucleophile behavior and the effect of microenvironmental, their practical decontamination or therapy technologies remain limited by such parameters as limited stability, low substrate selectivity, and incompatibility with the complex biological matrices. The newer development involving ionic‐liquid‐supported micelles adds chemical diversity to them but in no basic way imposes fundamental shortcomings of the type diffusion and compartmentalization limits. Having undertaken the conceptual and dynamic limits of micellar catalysis, the succeeding section addresses alternative organized media like cyclodextrins, liposomes, and polymeric or hybrid aggregates which merge molecular recognition with nucleophilic activity. These systems represent the new phase in the development of the detoxification of organophosphates in which supramolecular design and material engineering merge for the purposes enhancing selectivity, controlling reactivity, and moving toward practical applicability in the real world.

## 5 Supramolecular and Polymeric Platforms for Organophosphate Detoxification

The search for more effective environments for organophosphate (OP) degradation has gradually evolved beyond classical micellar catalysis toward structurally defined supramolecular and material‐based systems [103, 104]. In contrast to simple conventional surfactants, these assemblies provide a higher level of spatial organization and chemical selectivity, enabling not only catalytic acceleration but also molecular recognition, substrate encapsulation, and controlled release. Initial advances in this direction emerged from biomimetic inspiration: liposomes and cyclodextrins offered compartmentalized or host–guest environments capable of binding and transforming OP molecules under mild aqueous conditions. Liposomal systems added the option of sequestration of nucleophiles, enzymes, or reactivators in bilayer membranes to localize the reactive zone in a manner analogous to biological detoxification. Cyclodextrins, on the other hand, provided preorganized hydrophobic cavities that could selectively capture OP substrates or their surrogates, orienting them for nucleophilic attack by either encapsulated or appended functional groups. As research progressed, these supramolecular hosts were complemented by polymeric and hybrid materials, which extended the concept of confinement to solid or gel‐like matrices.

Functionalized polymers with oxime or hydroxamate pendant groups, cross‐linked hydrogels, and molecular imprinted polymers provided controllable microenvironments which combined chemical robustness with reusability and molecular selectivity in the uptake. Concurrently, inorganic supports such as silica, zeolitic, and metal–organic scaffolds provided solid or gel‐like frameworks with imperforate 3D structures of nanodomains and precise porosity for surface‐catalyzed or adsorptive detoxification. Taken together, all these systems signify the conceptual leap out of the reactivity enhancement entirely through local concentration effect towards the constructional engineering of the chemical microenvironment itself. The emphasis shifts towards molecular recognition realization in multistep reactivity with operational robustness under realistic conditions.

We begin this section by examining the use of liposomes as organized media for chemical decontamination with oximes. Their bilayer architecture and ability to encapsulate both hydrophilic and hydrophobic agents make them particularly attractive for delivering reactive species or sequestering toxic compounds in aqueous environments.

Ghosh and coworkers at the University of Raipur extended their long‐standing expertise in micellar catalysis to vesicular systems, investigating the catalytic efficiency of cationic liposomes formed from DODAC and DDAB in promoting the hydrolysis of organophosphate pesticides such as paraoxon, parathion, and fenitrothion [105]. In this study, oximes and hydroxamates were used as α‐active nucleophiles, and their reactivity was correlated with physicochemical parameters of the vesicular system, such as degree of vesicle formation, standard free energies of vesicle formation, adsorption, and vesicle formation per alkyl chain. Besides, it is clearly manifested that the minimum surface area per molecule increases with increasing the concentration of oximate and the reactivity towards OP pesticide also increases.

The vesicles showed superior catalytic efficiency to micelles, especially in the case of DODAC, and post‐reaction morphological changes were observed by SEM and TEM. This work represents a significant transition in Ghosh's work, applying his expertise in functionalized surfactants to the design of vesicles with emerging catalytic properties.

In a noteworthy contribution to this field, Pashirova et al. [106] developed cationic liposomes incorporating DHDHAB, a surfactant bearing a hydroxylated headgroup, to encapsulate 2‐PAM and enable its intranasal delivery in paraoxon‐poisoned rats. The formulation exhibited excellent colloidal stability, low hemolytic toxicity, and a high encapsulation efficiency (~90%). Most notably, intranasal administration of liposomal 2‐PAM achieved a 12% reactivation of cerebral AChE, in sharp contrast to the ineffectiveness of free 2‐PAM via the same route. These findings provide strong evidence for the potential of liposomes as delivery vehicles for oximes targeting the central nervous system and underscore the promise of noninvasive administration strategies in counteracting the neurotoxic effects of organophosphates. Moreover, it opens new avenues for reformulating conventional reactivators to broaden their therapeutic applicability.

Among supramolecular scaffolds, sulfonatocalix[4]arenes represent one of the most advanced small‐molecule platforms for OP detoxification. Early studies established that calix[4]arenes bearing a single hydroxamic acid can host cationic V‐type agents (e.g., VX) and promote selective P–S bond cleavage with half‐lives of only a few minutes under physiological conditions [107]. Subsequent structure–activity optimization revealed that introducing a second hydroxamic substituent along the rim markedly enhances activity, enabling sub‐minute detoxification rates for several V‐type agents and illustrating how cavity preorganization controls reactivity [108]. More recently, diversified calix[4]arene libraries have expanded this chemistry toward broader OP substrates, with clear SAR trends and, importantly, the first demonstrations of in vivo protection in murine OP‐poisoning models, positioning calixarenes as one of the most mature supramolecular families in this field [109].

**FIGURE 8:**
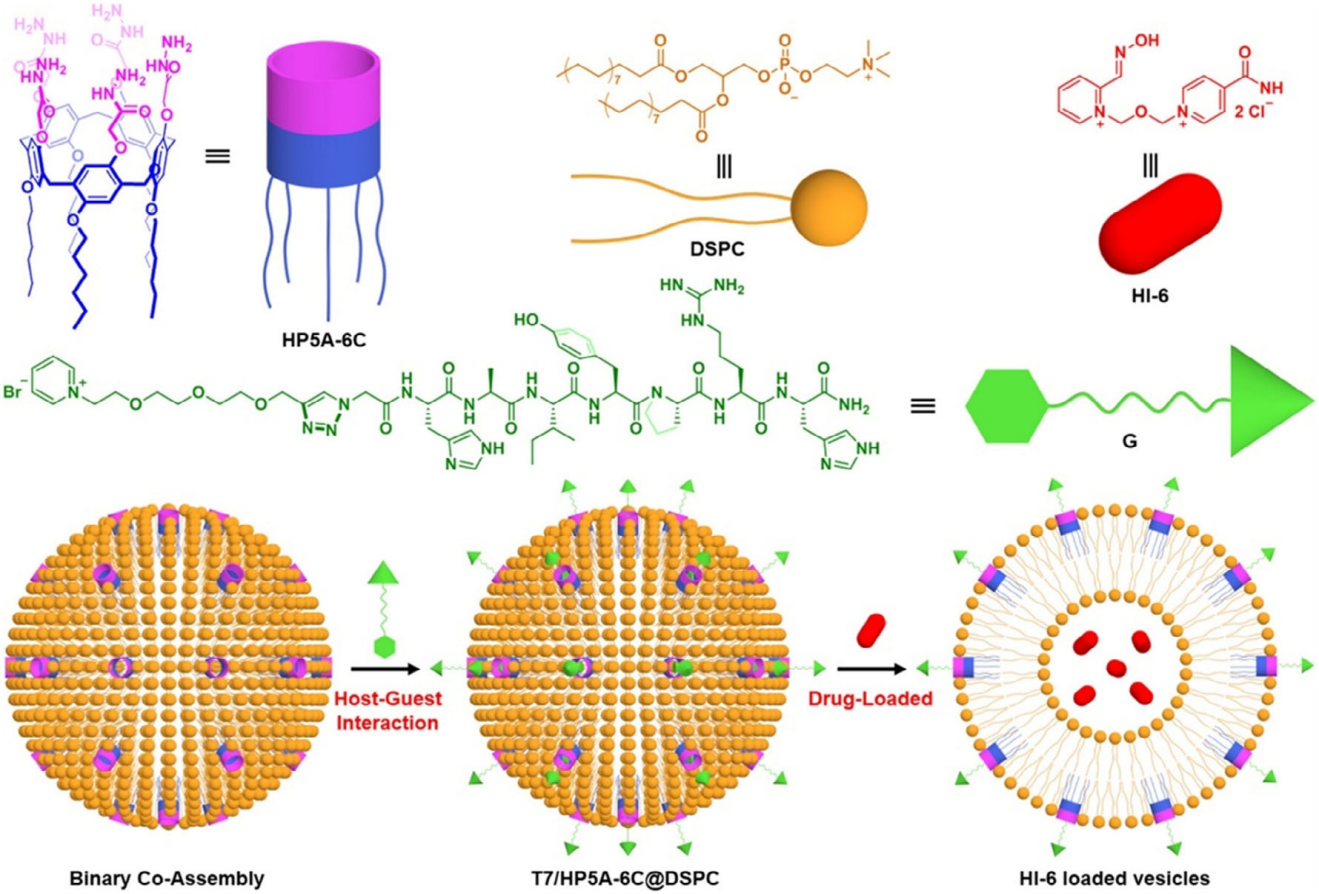
Chemical structures of HP5A‐6C, DSPC, HI‐6, and G, and schematic illustration of preparation of HI‐6‐loaded vesicles with brain‐targeting function. Reproduced from ref [110], with permission from American Chemical Society. Copyright 2024.

Zhang et al. [110] presented the most advanced and clinically relevant system in studies of liposomes with oximes. Using a supramolecular strategy, they designed liposomes functionalized with an amphiphilic pillar[5]arene (HP5A‐6C) and a T7 peptide targeting the transferrin receptor, which is overexpressed in the cerebral endothelium (see Figure 8). This system allowed HI‐6 to be encapsulated with 89.7% efficiency, maintaining structural stability for 6 weeks and showing low toxicity in vitro and in vivo. In paroxon‐poisoned mice, intravenous HI‐6‐loaded liposome administration doubled the survival rate in comparison with free HI‐6 (60% vs. 30%), retarded seizure onset time, and considerably diminished histological evidence of brain damage. This visionary work signifies the intersection of supramolecular chemistry, nanotechnology, and emergency pharmacology in setting a new standard in the targeted delivery of cholinesterase reactivators to the cerebral area.

While liposomal and vesicular systems have demonstrated considerable promise in enhancing the delivery and efficacy of oximes and catalytic agents, polymer‐based platforms offer complementary advantages in terms of structural versatility, tunable release kinetics, and functionalization capacity. Polymers can be engineered to respond to physiological stimuli, improve circulation time, and facilitate targeted delivery through covalent or supramolecular strategies. The following discussion reviews several recent journal articles that adopt a different approach to polymeric carriers for organophosphate detoxification, focusing on their design principles, encapsulation performance, and therapeutic outcomes in comparison to lipid‐based systems.

In 2010, Russell et al. [111] developed a multiagent platform combining chemical detoxification and antimicrobial activity. This paper presents a copolymer of dimethylacrylamide and methacrylate (DMAA‐MA), functionalized with 4‐pyridinium aldoxime moieties and halogens as counterions. This molecular design enables dual functionality: detoxification of organophosphorus compounds such as diisopropylfluorophosphate (DFP) via reactivation of inhibited acetylcholinesterase (AChE), and generation of reactive halogen species (I_2_, Br_2_) with biocidal properties. One of the oxime‐based reactivators was found to be released from the polymer matrix and successfully restored AChE activity. The described materials achieve simultaneous decontamination of both chemical and biological threats without mutual interference. Notably, the biocidal activity targeting biological agents may even enhance the efficiency of chemical detoxification.

**SCHEME 13:**
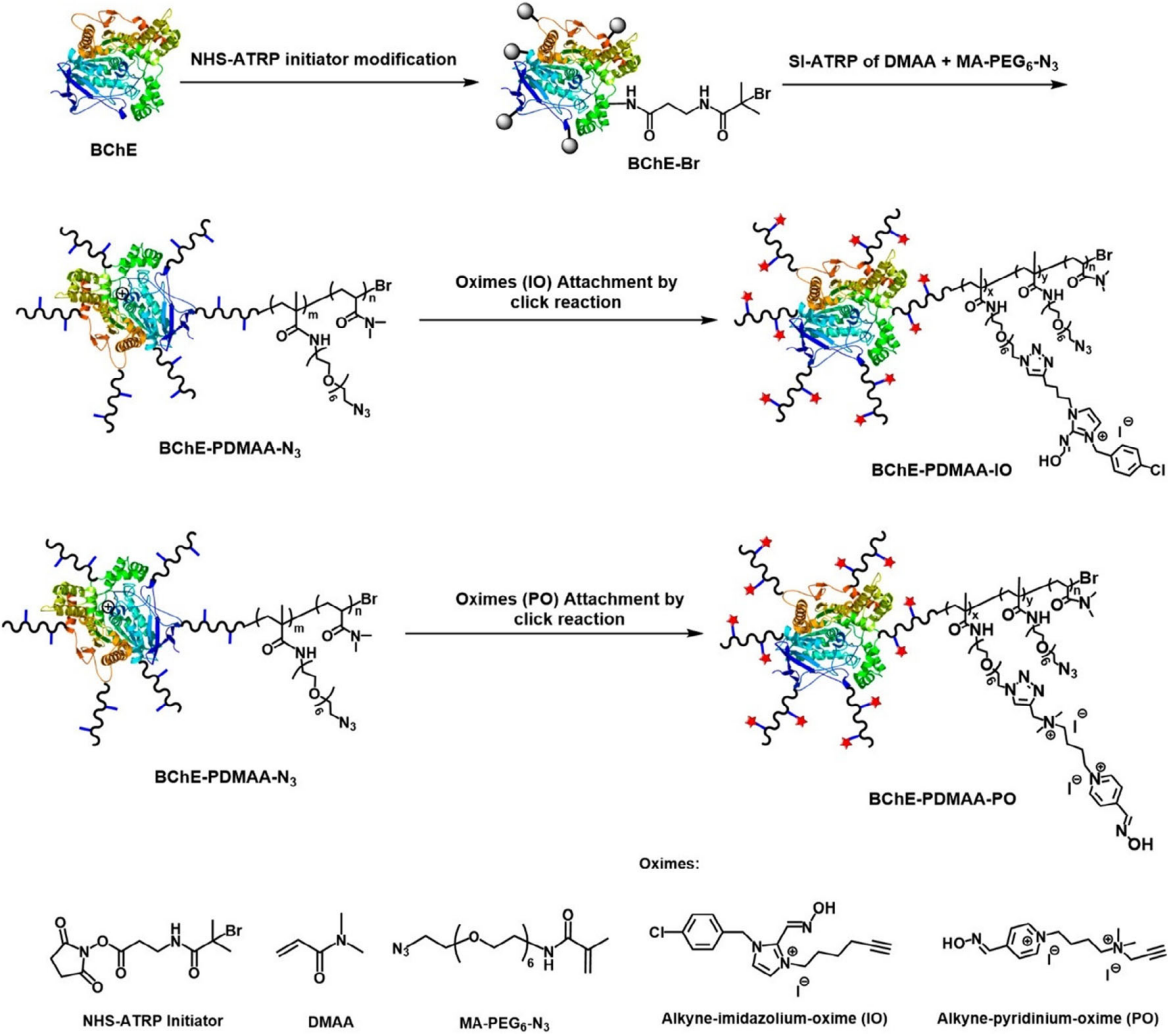
Synthesis of butyrylcholinesterase‐polymer‐oxime conjugates using atom‐transfer radical polymerization (ATRP) and “click” chemistry. Additional acronyms: N‐hydroxysuccinimide (NHS), N, N‐dimethylacrylamide (DMAA), N‐(20‐azido‐3,6,9,12,15,18‐hexaoxaicosyl)methacrylamide (MA‐PEG6‐N3), butyrylcholinesterase (BChE). Reproduced from ref [112], with permission from American Chemical Society. Copyright 2020.

In a different line of research, the same group introduces a new class of bioscavengers based on butyrylcholinesterase (BChE) conjugated with oxime‐functionalized polymers (Scheme 13) [112]. Bioscavengers are biological molecules, typically enzymes, that bind and neutralize organophosphorus compounds before they reach their targets in the nervous system; however, a detailed discussion of bioscavenger systems falls beyond the scope of this article. Nonetheless, using atom transfer radical polymerization (ATRP) and click chemistry, the authors grafted oxime‐containing copolymers onto BChE to create conjugates capable of self‐reactivation after inhibition by organophosphorus nerve agents. These conjugates showed prolonged enzymatic activity and could undergo multiple inhibition–reactivation cycles with fluorogenic VX and cyclosarin analogs. The reactivation efficiency correlated with oxime loading, and both intra‐ and intermolecular mechanisms contributed to detoxification. The conjugates also demonstrated protective effects and catalytic degradation of OPNAs, suggesting their utility as next‐generation bioscavengers for nerve agent decontamination.

Advances in polymer‐based materials have opened promising avenues for the decontamination of chemical warfare agents, particularly through the incorporation of nucleophilic functionalities such as oximates and halide ions. Marciano et al. (2011) demonstrated the catalytic degradation of VX using Amberlite IRA 900 F, a polystyrene‐divinylbenzene resin functionalized with ammonium fluoride groups [113]. Building on the concept of nucleophilic degradation, Hailing et al. introduced a novel acrylate copolymer, functionalized with 4‐pyridinium aldoxime groups covalently attached to the polymer backbone [114]. This oximated material achieved detoxification rates above 85% for GB, VX, and HD within minutes, with no formation of secondary toxic species. Mechanistic studies revealed a combination of nucleophilic substitution and second‐order Beckmann rearrangement, supported by chromogenic and spectrometric analyses.

Wong et al. described in 2020 [115] a library of dendrimer‐based nanoreactors designed for topical decontamination of organophosphates (OPs). The nanoreactors consist of poly(amidoamine) (PAMAM) dendrimers functionalized with oxime or hydroxamic acid groups and partially shielded with PEG chains. Screening revealed that several conjugates outperform Dekon 139 (the active component in reactive skin decontamination lotion) in hydrolyzing paraoxon and preventing skin penetration. These compounds exhibited catalytic activity via nucleophilic substitution reactions and demonstrated efficacy in both porcine skin and human epidermis models. Their outstanding ability to prevent paraoxon‐ethyl (POX) penetration is fundamentally linked to their enhanced reactivity in POX inactivation. In addition to their potent detoxifying performance, several lead conjugates showed favorable biocompatibility profiles in vitro, including lack of dermal permeation in porcine skin and compatibility with acetylcholinesterase (AChE) and human neuroblastoma cells. Further in vivo evaluations are currently underway, with a focus on immunogenicity and toxicological safety. While polymeric materials provide high local concentrations of reactive sites, cyclodextrins introduce a complementary approach based on molecular recognition and host–guest inclusion, offering a distinct mechanism for substrate activation and detoxification. In this context, the following studies focus on supramolecular scavengers, where β‐cyclodextrin derivatives act as host molecules that encapsulate organophosphorus agents and facilitate their degradation through strategically positioned reactive groups.

Building on this host–guest approach, Estour et al. [116] reported the development of a new generation of scavengers designed to neutralize OP compounds through inclusion complexation and subsequent nucleophilic attack. To this end, the authors synthesized a series of functionalized cyclo‐maltoheptaose (β‐cyclodextrin, β‐CD) derivatives bearing reactive groups designed to mimic enzymatic activity. Once encapsulated within the hydrophobic cavity of β‐CD, the OP compound becomes accessible to nucleophilic attack by the reactive moiety tethered to the cyclodextrin scaffold. The results demonstrated that all synthesized β‐CD derivatives were effective in detoxifying cyclosarin. Interestingly, the hydrolysis rate appears to be unaffected by the positional variation of the linker connecting the β‐CD core to the reactive moiety. Moreover, enantioselective hydrolysis of cyclosarin was observed in two of the tested compounds, highlighting their potential for stereospecific detoxification. The authors emphasize the relevance of β‐CD‐based architectures and propose further investigation into structure–activity relationships (SAR) to refine reactivity profiles and improve synthetic efficiency.

In a subsequent article, Kubik et al. [117] demonstrated that hydroxamic acid‐functionalized β‐CDs outperform oxime‐based analogs in degrading tabun. By arranging multiple hydroxamic acid groups along the CD cavity, they achieved half‐lives as low as 3 min for tabun detoxification at physiological pH and temperature. Mechanistic investigations revealed that the reaction proceeds via irreversible covalent modification of the scavenger, indicating a stoichiometric rather than catalytic process. Interestingly, glucose derivatives lacking the CD ring exhibited comparable activity, suggesting that the CD scaffold primarily serves as a multivalent support rather than an essential binding site.

**SCHEME 14:**
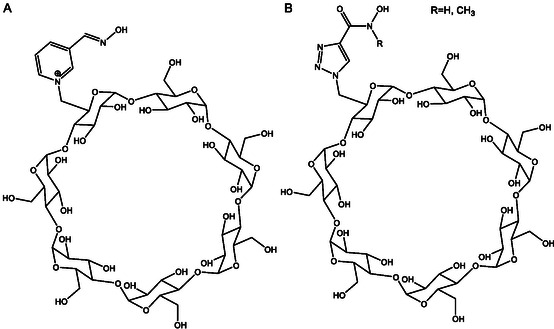
Structures of modified β‐CD 6‐(3‐oximepyridine)‐CD or 6‐OxP‐CD (A) and modified β‐CD with a hydroxamic acid substituent (B).

Building on the promising in vitro results of oxime‐functionalized cyclodextrins, Worek's group also provided the first in vivo demonstration of a cyclodextrin derivative acting as a small molecule scavenger against a nerve agent [118]. In guinea pigs exposed to cyclosarin, prophylactic administration of a β‐CD bearing a pyridinium oxime at the 6‐position prevented lethality and preserved brain acetylcholinesterase (AChE) activity. While erythrocyte AChE was not protected, the compound's ability to detoxify cyclosarin in circulation before CNS penetration marked a significant advance in nonenzymatic prophylaxis. In 2015, it was performed by the same research group a detailed in vitro study to elucidate the molecular mechanisms of cyclosarin detoxification by 6‐OxP‐CD (Scheme 14) [119]. Using stereoselective GC–MS and LC‐MS/MS, they showed that the compound preferentially eliminated the more toxic cyclosarin enantiomer. In contrast to unsubstituted β‐CD, the formation of tetrakis conjugates between organophosphorus (OP) compounds and 6‐OxP‐CD (enabled by the participation of the pyridinium oximate substituent) was demonstrated for the first time. The proposed reaction pathways are summarized in a schematic representation, offering the first mechanistic insights into OP detoxification by substituted cyclodextrins, which act as supramolecular scavengers with potential in vivo relevance. To further clarify the reaction pathways, analogous glucose derivatives were investigated by analogous derivatives bearing oxime or hydroxamic acid groups (Scheme 14) [120]. Their mechanistic study revealed that all nucleophiles tested, including oximes and hydroxamic acids, undergo irreversible chemical modification upon reaction with cyclosarin, tabun, and VX. For cyclosarin, oximes were phosphylated and then eliminated to form nitriles. Hydroxamic acids reacted with tabun in two ways: by forming stable phosphate esters or by undergoing a Lossen rearrangement with VX, producing urea derivatives. These findings confirmed that these compounds act stoichiometrically rather than catalytically, and that their detoxification mechanisms depend largely on the structure of the nerve agent or the nature of the nucleophile.

More recently, Valdez et al. [121] reported an elegant study in which the oxime‐functionalized cyclodextrin 6‐OxP‐CD was examined as a supramolecular scavenger for several nerve agents using combined ^31^P‐NMR experiments and MD/MM‐GBSA simulations. Their results showed that cyclosarin is readily neutralized through a highly favorable inclusion geometry, whereas soman adopts less reactive orientations within the cavity, leading to a more gradual detoxification. VX, in contrast, fails to engage in a productive inclusion complex, underscoring the importance of proper spatial pre‐organization for efficient reaction. Overall, this work highlights how inclusion geometry and steric alignment between the agent and the oxime govern the detoxification outcome, suggesting that fine‐tuning cavity size or nucleophile positioning may broaden the reactivity profile of cyclodextrin‐based scavengers.

**FIGURE 9:**
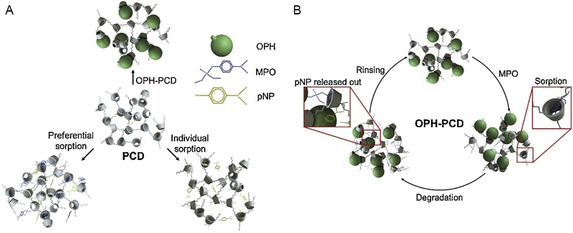
(A) Schematic illustration for PCD as a sorptive and regenerative system with the ability to be functionalized by adsorption of enzyme onto its structure. (B) Schematic illustration for OPH‐PCD as a stable self‐decontaminating bio‐catalytic system for sorption and degradation of organophosphates. Reproduced from ref [122] with permission from Elsevier. Copyright 2019.

In a significant evolution of cyclodextrin‐based detoxification platforms, Moon et al. [122] developed a hybrid material combining poly‐β‐cyclodextrin (PCD) with immobilized organophosphorus hydrolase (OPH), yielding a self‐decontaminating biocatalytic system. This composite, OPH–PCD, merges the sorptive capacity of PCD with the catalytic degradation power of OPH, targeting organophosphate pesticides such as methyl paraoxon (Figure 9). The PCD matrix, synthesized via crosslinking β‐cyclodextrin with hexamethylene diisocyanate, exhibited enhanced sorption for hydrophobic compounds. OPH immobilization onto PCD preserved enzymatic activity and enabled continuous hydrolysis of methyl paraoxon (MPO) into para‐nitrophenol (pNP) and phosphonic acid over 24 h in packed bed reactors. The system demonstrated excellent reusability and stability, maintaining catalytic function over four consecutive days and multiple sorption cycles. Importantly, the dual functionality of OPH–PCD offers a regenerative and environmentally friendly approach to nerve agent neutralization. The study highlights the potential of cyclodextrin‐based scaffolds not only as passive sorbents but also as active platforms for enzyme stabilization and deployment in field‐ready decontamination systems.

Cyclodextrin‐based systems have proven exceptionally versatile for the sorption and degradation of organophosphorus compounds, having progressed from basic host–guest configurations to multimodal platforms that incorporate enzymatic activity. Their biocompatibility, structural tunability, and ability to form inclusion complexes have made them attractive candidates for supramolecular detoxification strategies. While, as with micellar systems, the field has seen little conceptual innovation in the past 5 years, recent progress has been largely formulation tweaks or hybrid strategies with outstanding problems such as catalytic turnover and scalability untouched. By contrast, inorganic materials and nanostructured systems offer an intrinsically distinct organophosphate‐neutralization strategy with inherent catalysis and physicochemical stability. It has become interesting a new generation of detoxifying agents capable of rapid and often catalytic degradation under ambient conditions.

## 6 Detoxification Nanostructured and Inorganic Catalysts for Organophosphate Degradation

Nanostructured and inorganic materials are currently being studied as interesting supramolecular systems for the degradation of OP compounds showing promising results in both in vitro and in vivo applications. Their structural flexibility, high specific surface area, intrinsic catalytic activity, and physicochemical stability make them more desirable compared to micelles, vesicles, or cyclodextrins. Metal oxides, nanocomposites, and metal–organic frameworks are highlighted in recent research. Their stability and resilience have also conducted research on their incorporation into useful protective technologies, such as surface coatings, filters, and textiles. In this part of the review, we highlight some articles that investigate inorganic and nanoparticulate systems. How they work at the molecular level and how their performance compares in the context of detoxification.

In 2011, Hatton et al. introduced montmorillonite K‐10 clay functionalized with pralidoxime (PAM) and its zwitterionic oximate form (PAMNa) [123]. The study demonstrated that clay functionalized with an oximate compound is effective in degrading organophosphorus (OP) toxins, including both a pesticide and a chemical warfare agent analog. The synergistic interaction between the montmorillonite clay and 2‐PAM enhances their respective abilities to chemisorb and degrade OP compounds. This functionalized clay represents a straightforward and scalable strategy for developing reactive barrier materials and protective coatings. Notably, the incorporation of ClayPAMNa (known for its high binding efficiency toward OP agents) into elastomeric films, such as those used in gloves for military personnel and first responders, offers a potential solution to the well‐documented issue of secondary contamination in chemical agent‐resistant coatings. Upon exposure, these coatings may absorb a portion of the toxic agent, posing a risk of re‐emission even after decontamination. Furthermore, given that montmorillonite is listed in the U.S. FDA's Food Additive Database and 2‐PAM is an approved therapeutic agent, their combination via intercalation yields a biocompatible material suitable for applications such as reactive skin decontamination lotions and other detoxification formulations.

In a very different approach, the research group led by Yatsimirsky reported that coordination of oximate ligands to Zn(II) and Cd(II) ions dramatically enhances their esterolytic activity [124]. The resulting metal–oximate complexes cleaved 4‐nitrophenyl acetate (NPA) with rate constants exceeding two orders of magnitude above the reactivity limit of free oximates. This study also demonstrated that coordinating oximate anions to metal centers can effectively overcome the 'solvational imbalance’ surrounding the nucleophile, a key limitation previously highlighted by Terrier in the development of highly reactive oximate‐based nucleophiles [15, 16]. The reactivity of these metal–oxime complexes is highly sensitive to both the ligand architecture and the nature of the metal ion, likely due to solvation effects on the coordinated oxime group, although the underlying factors remain poorly understood. Remarkably, with an appropriately designed ligand, a moderately basic complex (pK_a_ ≈ 9) can exhibit esterolytic activity comparable to that of leading biocatalysts. Moreover, coordinated oximates are capable of operating under catalytic conditions, achieving high turnover rates.

**FIGURE 10:**
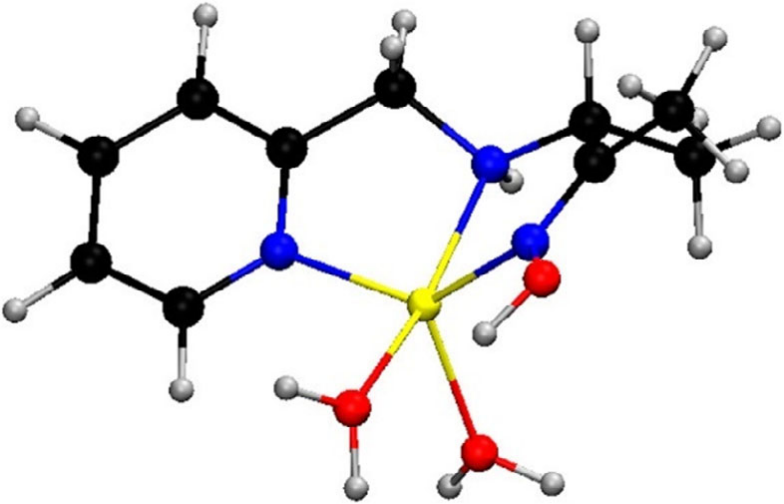
DFT‐optimized structure of [Zn(OxH)(H_2_O)_2_]^2+^ complexes. Reproduced from ref [125], with permission from American Chemical Society. Copyright 2017.

Following their previous work, Yatsimirsky and Gómez‐Tagle has gone a step further to exploit the catalytic potential of coordinated oximate nucleophiles. In a 2017 article, the authors provided a detailed mechanistic analysis of zinc(II) and cadmium(II) complexes of a tridentate oxime ligand [125]. These complexes showed high efficiency in cleaving triester compounds such as parathion, paraoxon, and 4‐nitrophenyldiphenyl phosphate (NPDPP). Kinetic results demonstrated that these complexes not only overcome the solvational imbalance that limits the reactivity of free oximates, but also exhibit unprecedented esterolytic activity, surpassing even that of natural esterases. Notably, the formation of a highly reactive [Zn(Ox)(OH)_2_]^−^ species (Figure 10) at high pH was confirmed both experimentally and computationally, and shown to be responsible for the exceptional catalytic performance. The study also revealed a shift in the rate‐determining step from nucleophilic attack (in free oximates) to leaving group departure (in metal complexes), providing a compelling explanation for the observed reactivity enhancement.

The same research group expanded their investigation to a broader family of newly synthesized tridentate and tetradentate oxime ligands [126]. Structural variations in the ligands, such as the size of the chelate rings and the total number of donor atoms, affect the stability and basicity of the resulting metal–oximate complexes, although their impact on reactivity is comparatively modest. Acetate ester cleavage proceeds via a truly catalytic pathway, with turnover numbers reaching up to 100, whereas phosphate ester hydrolysis remains a stoichiometric process. Notably, the reactivity of para‐substituted phenyl acetates was analyzed using Brønsted correlations, revealing a nonlinear relationship between the logarithm of the nucleophilic rate constant (log *k*_Nu_) and the pK_a_ of the conjugate acids of the leaving groups. This deviation suggests a shift in the rate‐determining step, from nucleophilic attack to leaving group expulsion, as the basicity of the leaving group increases within the pK_a_ range of 9.1–9.7, which is close to that of the coordinated oximes. The kinetic data support a mechanism involving electrophilic assistance from the metal center, which stabilizes the developing negative charge on the carbonyl oxygen in the transition state through a five‐membered cyclic intermediate. Both carboxylate and phosphate ester cleavage by coordinated oximate ligands occurs significantly faster than with free oximate anions. Importantly, the reactivity of the coordinated oximates exceeds that of highly basic free oximates by approximately two orders of magnitude. This enhancement is attributed to the suppression of the solvation imbalance effect, thanks to the reduced pK_a_ of the coordinated oxime, and to the stabilization of the transition state by the metal ion, which together account for the exceptional catalytic performance of these complexes. Together, these contributions from the Yatsimirsky and Gómez‐Tagle groups offer a compelling case for metal–oximate complexes as versatile and tunable nucleophilic platforms for organophosphorus detoxification. Notably, their approach is deeply rooted in the mechanistic framework established by Terrier, particularly in the use of Brønsted‐type correlations to probe the α‐effect and to rationalize deviations in reactivity through the concept of solvational imbalance. This continuity underscores the enduring relevance of Terrier's insights in guiding the design of efficient nucleophilic systems for OP degradation.

The 2025 Nobel Prize in Chemistry was jointly awarded to Susumu Kitagawa, Richard Robson, and Omar M. Yaghi for their pioneering work in the development of metal–organic frameworks (MOFs). MOFs are also systems that have been attempted for use in OP decontamination, but only a limited number of studies have explored them as carriers for oxime‐based antidotes [127]. For example, the titanium‐based MOF MIL‐125‐NH_2_ was able to efficiently adsorb and quickly release the antidote pralidoxime [128]. In another work, the iron‐based MOF MIL‐88B was evaluated for the sustained and long‐term delivery of the same oxime, demonstrating therapeutic efficacy in sarin‐poisoned mice following intragastric administration [129]. Despite these promising outcomes, the exceptional adsorption capacity of MOFs has not yet been fully leveraged in these systems.

**FIGURE 11:**
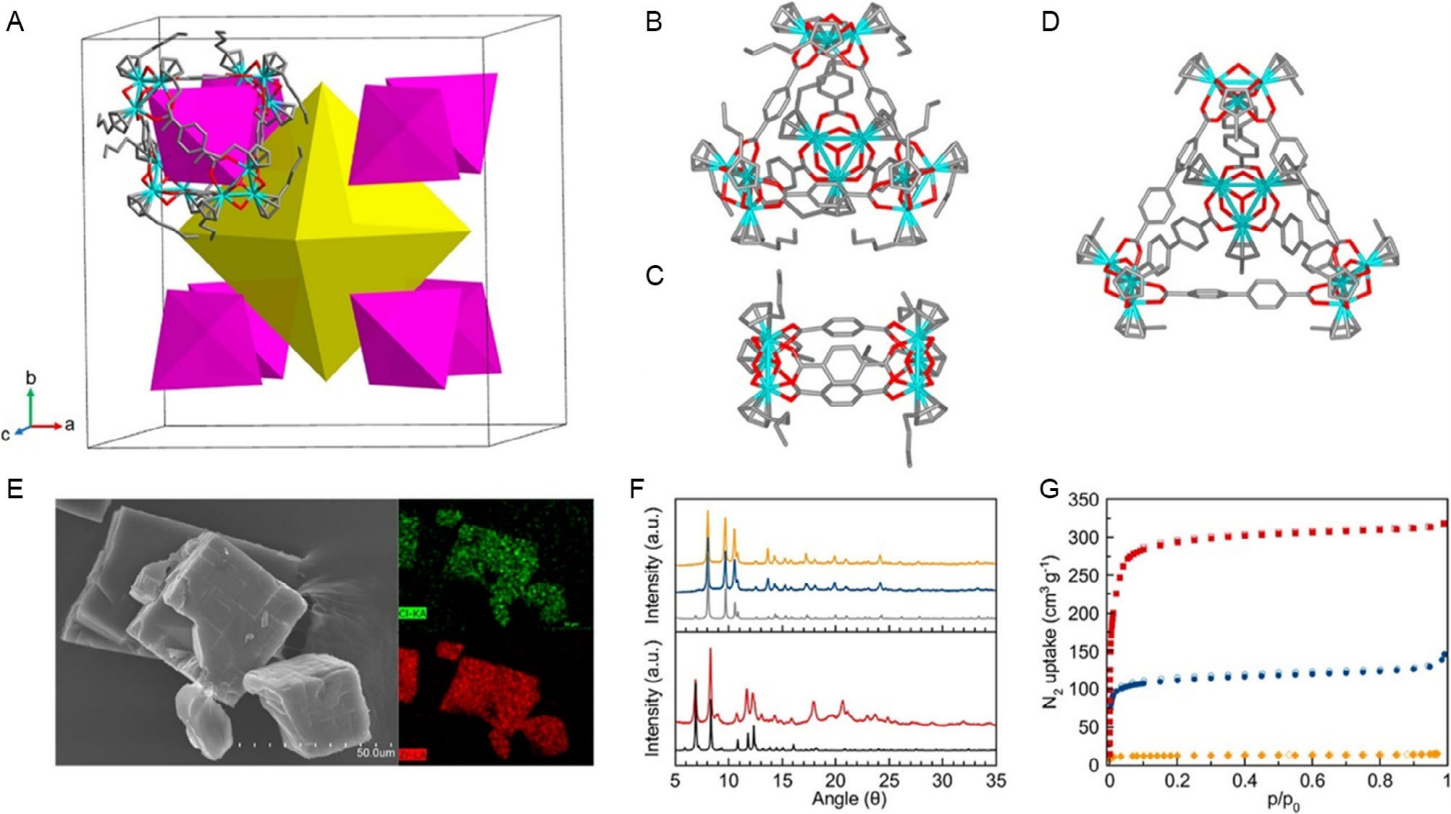
(A) Simplified crystal structure of Zr‐MOP‐1‐NH_2_: the fuchsia tetrahedra stand for the tetrameric cages and the yellow octahedron highlights the central cavity. (B) Tetrahedral cage of Zr‐MOP‐1‐NH_2_. (C) Dimeric isomer Zr‐MOP‐1′. (D) Tetrahedral cage of Zr‐MOP‐10, isoreticular to Zr‐MOP‐1‐NH_2_. Color code: zirconium, light blue; carbon, gray; and oxygen, red. Hydrogens and disordered NH_2_ residues are not depicted for clarity. (E) SEM–EDX images of Zr‐MOP‐1; color code: Cl, green and Zr, red. (F) On the top: comparison between the observed PXRD patterns of Zr‐MOP‐1 (blue trace) and Zr‐MOP‐1‐NH_2_ (yellow trace) and the simulated PXRD pattern of Zr‐MOP‐1‐NH_2_ (gray trace). On the bottom: experimental Zr‐MOP‐10 (red trace) and simulated Zr‐MOP‐10 PXRD patterns (black trace). (G) N_2_ adsorption isotherms at 77 K for Zr‐MOP‐1‐X (X = H and NH_2_, blue circles and yellow diamonds, respectively) and Zr‐MOP‐10 (red squares). Open symbols denote desorption. Reproduced from ref [130]. Licensed under CC‐BY 4.0.

More recently in an interesting article Carmona, Maldonado et al. developed a zirconium‐based metal–organic framework (MOF‐808) functionalized with the neutral oxime RS69N, yielding a hybrid material with dual functionality for organophosphate (OP) detoxification (Figure 11) [130]. The system combines sustained oxime release with efficient sorption of diisopropylfluorophosphate (DIFP), a nerve agent simulant. The MOF‐808 matrix, characterized by high porosity and accessible Zr(IV) sites, was loaded with RS69N via solid–liquid impregnation, achieving a drug payload of 5.2 ± 0.9 oxime molecules per MOF unit. In simulated physiological media, the material released RS69N gradually over 24 h, reaching 37.5% cumulative release. The released oxime retained full reactivation capacity against DIFP‐inhibited acetylcholinesterase (AChE), confirming its therapeutic viability. Simultaneously, the hybrid material removed 95% of DIFP from solution within 24 h, following pseudo‐second‐order kinetics. Importantly, the material reduced AChE inhibition by DIFP from 63.5% to 13.7%, demonstrating a significant detoxifying effect. This study exemplifies the potential of MOF‐based systems to integrate oxime delivery and OP capture in a single platform, offering a promising strategy for systemic detoxification with controlled pharmacokinetics and reduced toxicity.

Following their previous work with zirconium‐based metal–organic frameworks (Zr‐MOFs), the research team went on to synthesize and characterize zirconium metal–organic polyhedra (Zr‐MOPs), demonstrating their potential through a proof‐of‐concept application [130]. While Zr‐MOFs constructed from [Zr_6_O_4_(OH)_4_] clusters have been extensively studied for OP capture and hydrolysis due to their chemical robustness and Lewis acidity, their extended framework nature, unless downsized to the nanoscale, limits their suitability for biomedical applications. In contrast, Zr‐MOPs, which can be viewed as discrete molecular analogs of MOFs, offer both solid‐state stability and solution processability, making them attractive candidates for drug delivery. In this study, the authors synthesized robust tetrahedral cages decorated with lipophilic alkyl chains and featuring internal cavities of approximately 9.8 and 10.7 Å. These Zr‐MOPs were shown to effectively capture the organophosphate simulant diisopropylfluorophosphate (DIFP) and simultaneously host and release the AChE reactivator pralidoxime (2‐PAM). The resulting host–guest assemblies enabled sustained release of 2‐PAM under simulated physiological conditions, leading to measurable reactivation of DIFP‐inhibited acetylcholinesterase. Furthermore, incorporation of the Zr‐MOPs into phosphatidylcholine liposomes yielded biocompatible formulations that were nonneurotoxic, as confirmed by neuroblastoma cell viability assays.

Recent research has also focused on nanostructured materials, in which diffusion and reactivity operate on similar scales. Nanoparticle‐based catalysts function through reaction mechanisms that do not require nucleophilic activators. Therefore, they enable continuous catalytic detoxification under environmental conditions. Their versatility also allows them to be incorporated into commonly used materials, and in the near future they will become standard components in protection and decontamination technologies.

If we look back at some of the most notable works published in the last 20 years, we can start by considering the work of Bromberg and Hatton [131]. They introduced magnetite (Fe_3_O_4_) nanoparticles functionalized with oxime groups, either via 2‐pralidoxime (PAM) or a polymeric analog, as recyclable catalysts for organophosphate (OP) hydrolysis. These nanoparticles catalyzed the degradation of diisopropyl fluorophosphate (DFP) at neutral pH, achieving hydrolysis rates comparable to copper(II) chelates, but with the added advantage of magnetic recoverability. The oxime groups, immobilized on the nanoparticle surface, acted as nucleophilic centers, enhancing the hydrolysis rate by 2–3 orders of magnitude over spontaneous degradation. The system followed Michaelis–Menten kinetics, and PAM‐modified particles showed higher catalytic efficiency than PAM alone. Importantly, the particles retained their activity after multiple recovery cycles using high‐gradient magnetic separation, demonstrating excellent reusability and stability.

Solid lipid nanoparticles (SLNs) are promising nanocarriers for crossing the blood‐brain barrier (BBB) in drug delivery to the central nervous system (CNS). In a 2017 study, Zakharova, Masson et al. developed nanoparticles loaded with pralidoxime (2‐PAM) [132] Despite the permanent positive charge of 2‐PAM, which typically limits its ability to cross the BBB, the SLNs achieved a 15% reactivation of cerebral acetylcholinesterase (AChE) in rats exposed to paraoxon. The nanoparticles exhibited high encapsulation efficiency (~90%), good colloidal stability, and extended the plasma half‐life of 2‐PAM by a factor of six compared to its free form. Moreover, pretreatment with SLNs significantly reduced mortality in rats subjected to lethal doses of paraoxon. Given that montmorillonite is listed in the U.S. FDA's Food Additive Database and 2‐PAM is an approved drug, their combination through intercalation offers a biocompatible platform suitable for applications such as reactive skin decontamination lotions and other detoxification formulations.

Continuing their in vivo investigations with nanoparticle‐based delivery systems, Pashirova's group developed PEGylated solid lipid nanoparticles encapsulating pralidoxime chloride (2‐PAM), using DSPE‐PEG_2000_ as the surface modifier [133]. This nanoformulation was designed to enhance BBB penetration and improve pharmacokinetic performance. A pharmacokinetic study was conducted on plasma and brain using nanoparticles 2‐PAM‐DSPE‐PEG_2000_‐SLN. The experimental technique used for the quantitative determination of 2‐PAM in the biological compartments was high‐performance liquid chromatography tandem mass spectrometry with atmospheric pressure chemical ionization by multiple reaction monitoring mode (HPLC‐APCI‐MS). Intravenous administration of 2‐PAM‐DSPE‐PEG2000‐SLNs nanoparticles was performed. The experimental results showed an increase in the reactivation of human cerebral acetylcholinesterase inhibited by paraoxon of up to 36%. This significant result was one of the first examples where such a high reactivation value was achieved. Studies based on this type of nanoparticle need to continue evolving, as they offer room for improvement for their possible pharmaceutical use as antidotes in OP poisoning.

In another in vivo study, Liu et al. developed nanoparticles loaded with the oxime HI‐6 [134]. The system was constructed by conjugating the cyclic peptide c(RGDyK) to Pluronic P85, which, together with poly(butyl cyanoacrylate) (PBCA), served as the carrier matrix. This nanodrug delivery system has the advantages of active central targeting. In an in vitro BBB model, the nanoparticles exhibited a fourfold increase in penetration compared to free HI‐6, and in vivo experiments confirmed enhanced accumulation in the brains of treated mice. In poisoned animals, the formulation achieved a 19.3% reactivation of brain AChE, significantly higher than that obtained with free HI‐6 or without peptide modification. The system combines P‐glycoprotein efflux inhibition, active brain targeting, and favorable biocompatibility, positioning it as a promising strategy for the treatment of central nervous system poisoning by nerve agents.

**SCHEME 15:**
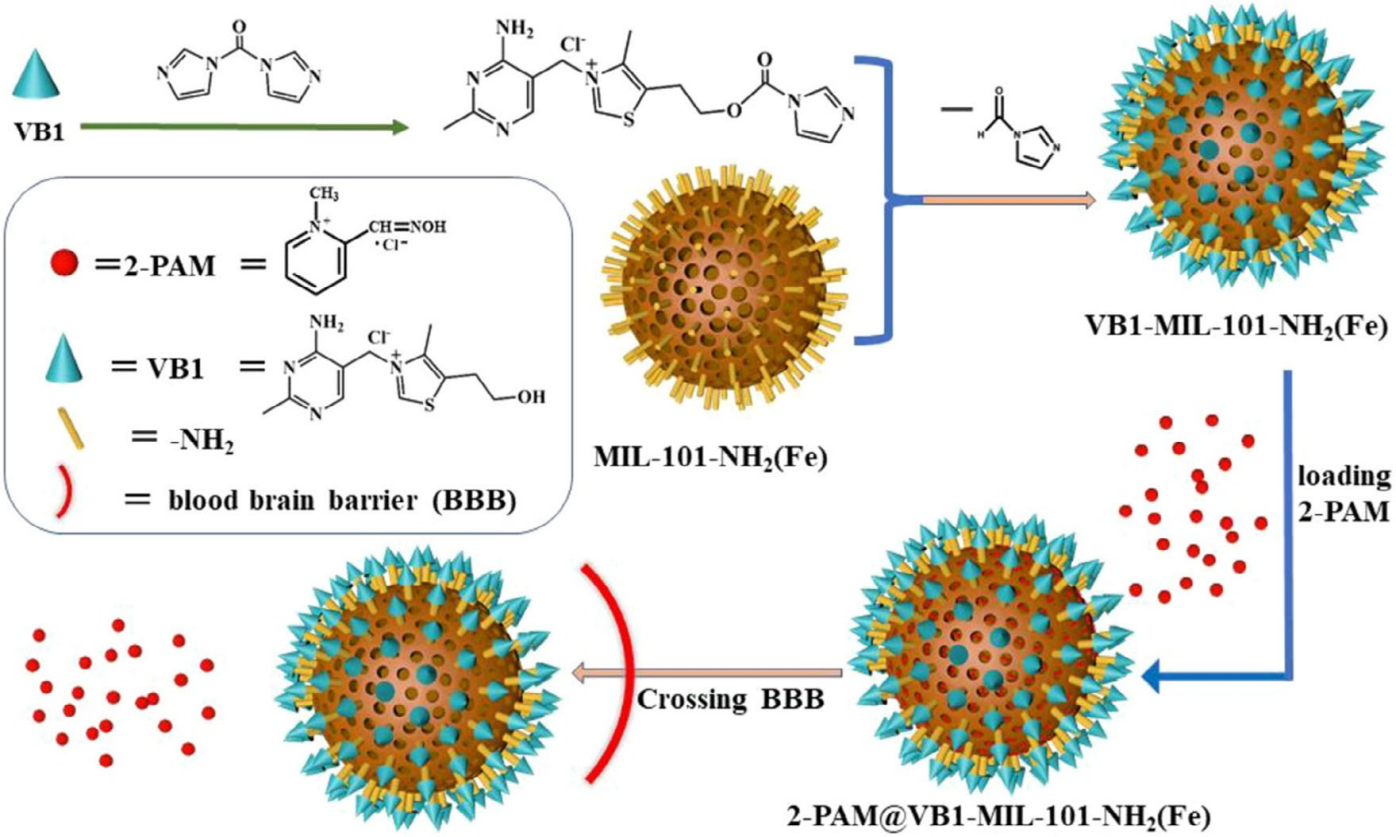
Schematic diagram of the preparation of 2‐PAM@VB1‐MIL‐101‐NH2(Fe) and targeted and sustained drug release across the blood–brain barrier. Reproduced from ref [135], with permission from American Chemical Society. Copyright 2023.

Metal‐organic frameworks (MOFs) can be prepared as nanoparticles. Zhou, Zhang, Zhong et al. prepared 2‐PAM@VB1‐MIL‐101‐NH2(Fe) (Scheme 15), a composite drug with an external surface modified with thiamine transporter (VB1) that can specifically bind to the surface of the blood−brain barrier and encapsulated 2‐PAM internally [135]. The composite exhibited a drug loading capacity of 14.8% and particle size around 100 nm. In vitro release studies showed pH‐dependent behavior, with up to 77.5% release at pH 4, and sustained release over 72 h. In vivo experiments in sarin‐poisoned mice demonstrated that the formulation significantly improved acetylcholinesterase (AChE) reactivation in blood (42.7% at 72 h) and brain tissue (up to 88.9%), outperforming nontargeted controls. Zebrafish and mouse models confirmed the composite's ability to cross the BBB, attributed to VB1‐mediated targeting and passive diffusion. Overall, the study presents 2‐PAM@VB1‐MIL‐101‐NH_2_(Fe) as another promising platform for middle‐ and late‐stage treatment of nerve agent poisoning, combining targeted delivery, sustained release, and effective central AChE reactivation. Further optimization and pharmacokinetic studies are suggested to advance its therapeutic potential.

**FIGURE 12:**
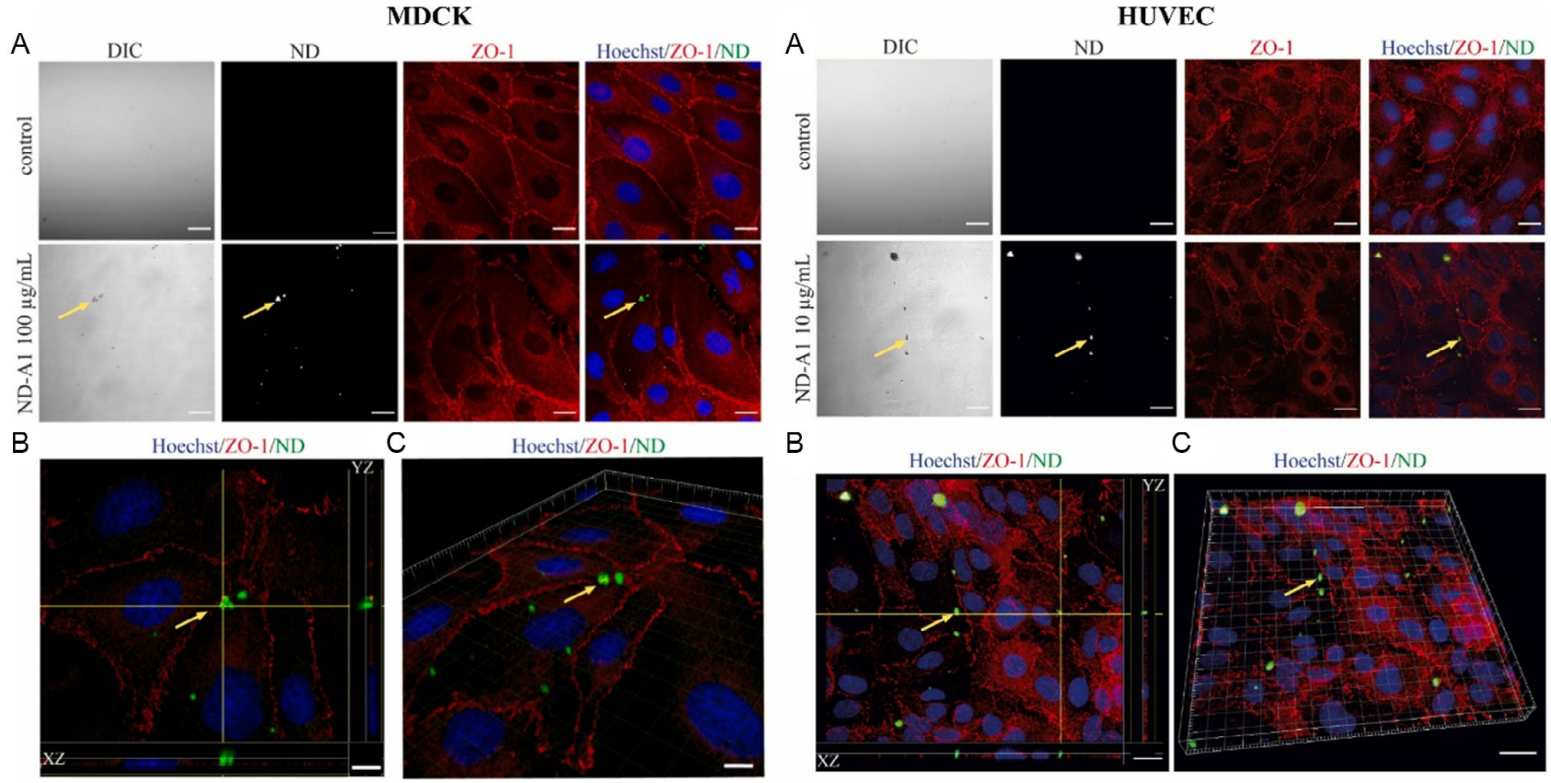
Entry of ND‐A1 into MDCK (left panel) and HUVEC (right panel) cells. (A) Confocal microscopy images show ND‐A1 uptake in MDCK (left) and HUVEC (right) cells. The top panel presents untreated controls, while the bottom panel shows MDCK cells treated with 100 μg/mL ND‐A1 for 3 h and HUVEC cells treated with 10 μg/mL ND‐A1 for 1 h. Cells were fixed and stained with ZO‐1 (red, cell junctions) and Hoechst 33 342 (blue, nuclei). ND‐A1 exhibits intrinsic fluorescence and appears green, visible through both DIC and 488 nm laser excitation. Merged images highlight internalized ND‐A1 (yellow arrows). (B) Orthogonal views from the z‐stack confirm intracellular localization of ND‐A1 (yellow arrows). (C) 3D rendering further visualizes ND‐A1 inside the cells, reinforcing its internalization. All images were processed using Imaris software. Scale bars: 20 μm. Reproduced from ref [136], with permission from Elsevier. Copyright 2025.

In 2025, Mochalin, Karpichev et al. introduced a novel strategy for delivering quaternary oxime antidotes across the blood–brain barrier using functionalized detonation nanodiamonds (NDs), i.e., nanodiamonds obtained by detonation synthesis [136]. Three oxime‐functionalized nanodiamond conjugates were synthesized starting from highly pure ND‐COOH. The functional groups were built using PEG_3_‐diamine as a spacer and 4‐oximinomethylpyridine as the active moiety. These were further modified with haloacyl halides containing linkers of different lengths (*n* = 1, 2, 3), resulting in ND‐A1, ND‐A2, and ND‐A3. The final conjugation to the nanodiamonds was achieved using N, N′‐carbonyldiimidazole as a coupling agent. Notably, ND‐A1 demonstrated good permeability in BBB surrogate models such as Madin‐Darby Canine Kidney (MDCK) cells and Human Umbilical Vein Endothelial Cells (HUVEC) cells, surpassing conventional oximes like obidoxime in terms of brain penetration. Confocal microscopy revealed that the nanoparticles were efficiently taken up by cells without causing significant toxicity or disturbing the integrity of tight junctions at low concentrations (Figure 12). While the ability to reactivate the enzyme remains moderate, the findings underscore the potential of nanodiamonds as innovative carriers for delivering antidotes directly to the central nervous system. Future improvements may arise from optimizing the oxime structure and nanoparticle surface chemistry to enhance dispersion and therapeutic efficacy.

Together, these studies showcase the range of nanoparticle platforms, including lipid‐based carriers and dendrimers, MOF‐based nanozymes, and carbon nanomaterials, in addressing key challenges in OP detoxification. Their modularity, biocompatibility, and capacity for targeted delivery position them as valuable tools for next‐generation antidote development.

Inorganic and hybrid platforms such as MOFs, MOPs, solid lipid nanoparticles, and functionalized nanodiamonds have become the most widely used and versatile tools for biomedical applications in organophosphate poisoning. Their adaptability to both in vitro and in vivo contexts, combined with tunable drug release, biocompatibility, and capacity for blood–brain barrier penetration, makes them exemplary models of what modern antidotal strategies should aim for. The early conceptual contributions of supramolecular chemistry should not be overlooked, even if the traditional systems‐like micelles or cyclodextrins no longer offer any conceptual novelty today, and mechanistic studies have provided deep insights into reaction pathways and limitations. Thus, the grounds for nucleophilic reactivity, solvation effects, and α‐effect, as pioneered by Buncel and Terrier, are still valid in the design of next‐generation therapeutic materials. The focus has currently shifted from the mechanistic exploration to translational optimization, and advanced materials represent indeed the forefront of what is achievable in the targeted, effective, and sustained treatment of neurotoxicant exposure.

## 7 Summary and Outlook

This review follows the trajectory of oxime‐based detoxification research from the mechanistic insights by Terrier to today's integration of synthetic chemistry, materials science, and computational design. Current strategies increasingly rely on nanoscale architectures, such as metal–organic frameworks and hybrid nanocomposites, that combine catalytic efficiency with biocompatibility. Moving forward, predictive modeling and mechanistic clarity will be essential to create adaptive platforms for both environmental and biomedical applications. From α‐effect chemistry to the era of smart nanomaterials, the next breakthroughs will emerge at the interface of computation and catalysis.

## Conflicts of Interest

The author declares no conflicts of interest.
